# Identification of Caspase Cleavage Sites in KSHV Latency-Associated Nuclear Antigen and Their Effects on Caspase-Related Host Defense Responses

**DOI:** 10.1371/journal.ppat.1005064

**Published:** 2015-07-28

**Authors:** David A. Davis, Nicole E. Naiman, Victoria Wang, Prabha Shrestha, Muzammel Haque, Duosha Hu, Holda A. Anagho, Robert F. Carey, Katharine S. Davidoff, Robert Yarchoan

**Affiliations:** HIV and AIDS Malignancy Branch, Center for Cancer Research, National Cancer Institute, Bethesda, Maryland, United States of America; University of North Carolina at Chapel Hill, UNITED STATES

## Abstract

Kaposi’s sarcoma-associated herpesvirus (KSHV), also known as human herpesvirus-8, is the causative agent of three hyperproliferative disorders: Kaposi’s sarcoma, primary effusion lymphoma (PEL) and multicentric Castleman’s disease. During viral latency a small subset of viral genes are produced, including KSHV latency-associated nuclear antigen (LANA), which help the virus thwart cellular defense responses. We found that exposure of KSHV-infected cells to oxidative stress, or other inducers of apoptosis and caspase activation, led to processing of LANA and that this processing could be inhibited with the pan-caspase inhibitor Z-VAD-FMK. Using sequence, peptide, and mutational analysis, two caspase cleavage sites within LANA were identified: a site for caspase-3 type caspases at the N-terminus and a site for caspase-1 and-3 type caspases at the C-terminus. Using LANA expression plasmids, we demonstrated that mutation of these cleavage sites prevents caspase-1 and caspase-3 processing of LANA. This indicates that these are the principal sites that are susceptible to caspase cleavage. Using peptides spanning the identified LANA cleavage sites, we show that caspase activity can be inhibited *in vitro* and that a cell-permeable peptide spanning the C-terminal cleavage site could inhibit cleavage of poly (ADP-ribose) polymerase and increase viability in cells undergoing etoposide-induced apoptosis. The C-terminal peptide of LANA also inhibited interleukin-1beta (IL-1β) production from lipopolysaccharide-treated THP-1 cells by more than 50%. Furthermore, mutation of the two cleavage sites in LANA led to a significant increase in IL-1β production in transfected THP-1 cells; this provides evidence that these sites function to blunt the inflammasome, which is known to be activated in latently infected PEL cells. These results suggest that specific caspase cleavage sites in KSHV LANA function to blunt apoptosis as well as interfere with the caspase-1-mediated inflammasome, thus thwarting key cellular defense mechanisms.

## Author Summary

Upon infecting a target cell, viruses must be able to overcome cellular defense responses to survive. Two of the most important cellular defense responses against viruses are apoptosis and the inflammasome, a component of the innate immune response. Apoptosis, a programmed cell death, functions to limit the spread of viruses by destroying the infected cell while innate immune responses control viral infections through other means. Both apoptosis and the inflammasome are mediated by caspases. However, many viruses are known to encode proteins that block, suppress or delay caspase activity following cellular infection in order to block cell death and interfere with the inflammasome. We show that LANA undergoes caspase-dependent cleavage in Kaposi’s sarcoma associated herpesvirus (KSHV)-infected cells, especially when exposed to oxidative stress. Through peptide, sequence and mutational analysis, we identified two sites for caspase cleavage in KSHV LANA, one in the N-terminal region and the other in the C-terminal region. Using synthetic peptides of these cleavage sites, we show that the C-terminal site can inhibit cleavage of poly (ADP-ribose) polymerase and enhance cellular survival. Furthermore, we demonstrate that this synthetic peptide inhibits the inflammasome response as evidenced by decreased interleukin-1beta (IL-1β) production. Mutation of these cleavage sites in LANA leads to a significant increase in the inflammasome response indicated by increased IL-1β production compared to wild-type LANA. Taken in total, these results provide evidence that these cleavage sites in LANA participate both in delaying apoptosis and blunting aspects of the innate immune response. These studies provide new insights into the mechanisms by which KSHV obviates the cellular defense responses that are activated following virus infection.

## Introduction

It is well established that most viruses have evolved mechanisms to thwart cellular defense responses, including programmed cell death (apoptosis) and the inflammasome, a component of the innate immune response [1–6]. Kaposi’s sarcoma-associated herpesvirus (KSHV), the causative agent of Kaposi’s sarcoma, primary effusion lymphoma, and multicentric Cattleman’s disease, is no exception. In most infected cells, KSHV is present predominantly in a latent state [7,8] and during latency KSHV expresses a number of genes that play pivotal roles in thwarting apoptosis and other cellular defense responses. The KSHV gene product vFLIP can inhibit apoptosis by preventing death receptor activation [9–12]. Also, KSHV vIRF-3 and latency-associated nuclear antigen (LANA) can prevent cell death induced through p53 activation [4,13]. In addition to these genes, the KSHV-encoded miRNAs miR-K12-1, 3 and 4-3p of KSHV inhibit the production of caspase-3, a key mediator of apoptotic cell death [14]. During lytic activation, KSHV expresses other genes that also function to maintain cell survival, including vBcl-2, which inhibits the intrinsic apoptotic pathway; vIAP, which inhibits BAX; and kbZIP, which inhibit cellular p53 [4]. In addition to these numerous mechanisms designed to prevent or delay apoptosis, KSHV also expresses latent and lytic genes that enable infected cells to evade the immune system. These include vFLIP and vIRF-1, which regulate MHC I expression [3,15]; LANA which evades MHC I peptide processing [16]; and ORF63, a lytic protein which blocks inflammasome activation and subsequent activation of caspase-1 [17]. With this multi-factorial system of defenses, KSHV is able to survive and proliferate in a number of different cell types.

Herpesviruses and the proteins they encode often induce oxidative stress upon infection, leading to the accumulation of oxidized proteins [18,19,20,21]. KSHV-infected cells generate reactive oxygen species [22], and KS tumors are under a state of chronic oxidative stress as indicated by increased expression of xCT, a receptor induced by oxidative stress that is used by cells to increase glutathione levels [23]. Oxidative stress can induce apoptosis and KSHV reactivation from latency [24,25], and it is advantageous for KSHV to control oxidative stress-induced apoptosis during latent and lytic infection, which could otherwise lead to the destruction of the virally-infected cells [26].

While studying the role of oxidative stress in KSHV infection, we discovered that LANA undergoes cleavage by caspases. Through peptide, sequence, enzyme, and mutational analysis we identified two bona fide caspase cleavage sites in LANA. We provide evidence that these sites function as caspase substrate decoys in order to delay or inhibit apoptotic events and blunt inflammasome activity. LANA is an abundant protein in infected cells, and it thus has the potential to inhibit these caspase-mediated defense responses. This process may be critical in contributing to the evasion of apoptosis and the inflammatory response, allowing for survival and dissemination of KSHV.

## Results

### Identification of putative caspase cleavage sites in KSHV LANA

**Fig 1:**
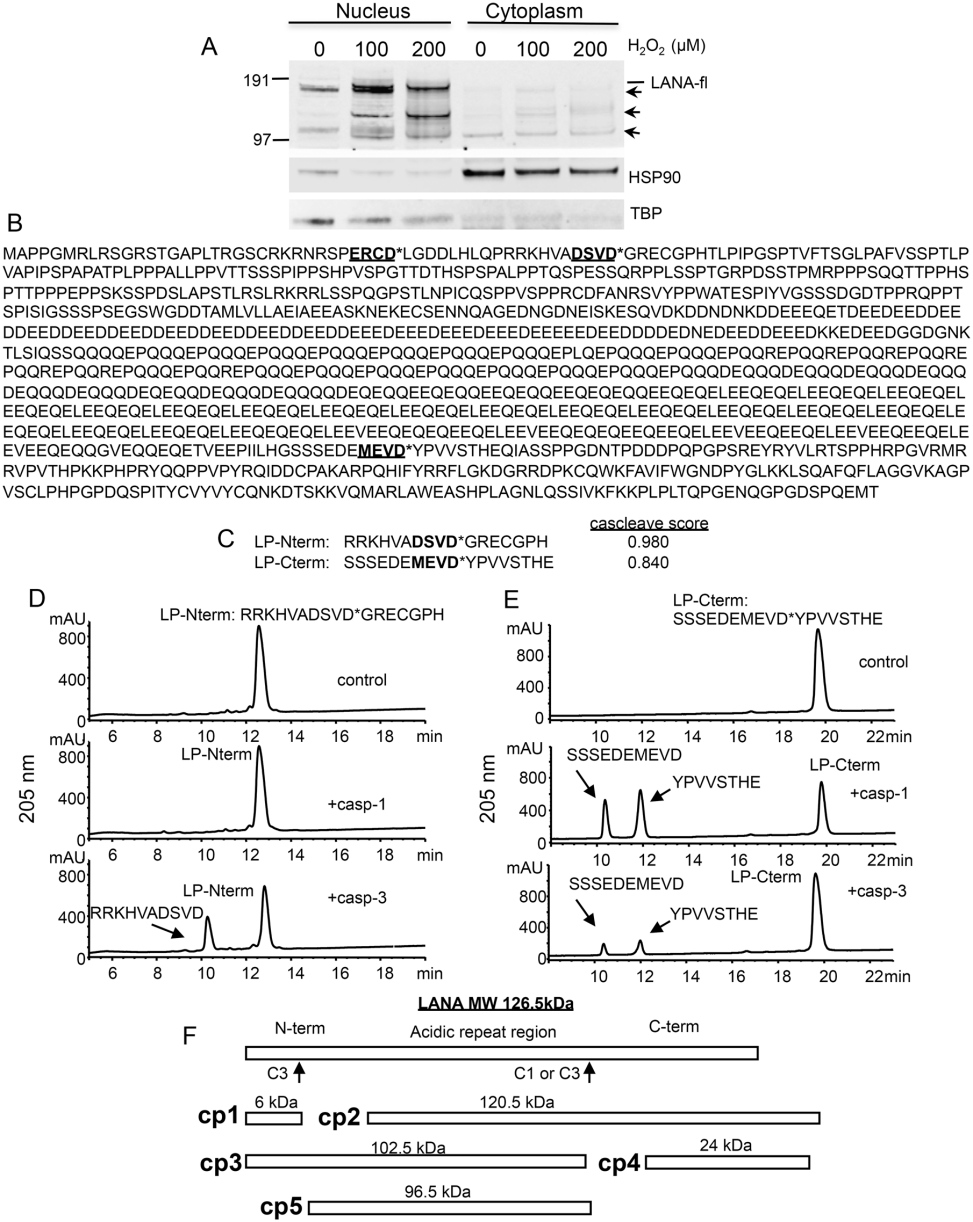
Changes in KSHV LANA following treatment of BCBL-1 cells with varying concentrations of H_2_O_2_ and sequence analysis of LANA identifying potential caspase cleavage sites. (A) BCBL-1 cells were cultured in media at 500,000 cells per ml to which was added H_2_O_2_ at 100 and 200 μM (diluted into PBS) or PBS as a control for 20 hrs. Nuclear and cytoplasmic extracts (10 μgs per sample) were prepared and analyzed by western blot for LANA using a monoclonal antibody that is directed towards the C-terminal region of LANA (Leica), and with antibodies directed toward TBP (rabbit) and HSP90 (mouse). Blots were then incubated with appropriate secondary antibodies (goat anti-mouse or rabbit IR800 secondary antibody) and analyzed using the LiCor system. Full length LANA (LANA-fl) and three faster migrating forms of LANA (arrows) are indicated. Molecular weight markers in kDa are indicated to the left of the blot. Full length LANA (calculated molecular weight of 126 kDa based on the amino acid sequence) migrates at approximately 165 kDa in the Nupage LDS gel system. HSP90 is shown as a cytoplasmic loading control and TBP as a nuclear loading control. (B) The 1095 LANA amino acid sequence translated from the KSHV genome derived from BCBL-1 cells (Genbank U93872) was analyzed for potential caspase cleavage sites utilizing the caspase webserver Cascleave (http://sunflower.kuicr.kyoto-u.ac.jp/~sjn/Cascleave/webserver.html). Two sites with high probability scores were located within the N-terminal domain of LANA, and one was located in the C-terminal domain. Shown in bold and underlined are these three putative caspase cleavage sites; cleavage by caspases is predicted to occur at the carboxyl side of aspartate as indicated by an asterisk (*). (C) The two peptides that were found to be cleaved by caspase-1 and/or -3 are shown and denoted as LP-Nterm and LP-Cterm (with Cascleave scores of 0.980 and 0.840, respectively). (D) Reverse phase HPLC of 1mM LP-Nterm (MW 1919 Da) following treatment with PBS control (top panel), caspase-1 at 2.5 units/μl (middle panel), or caspase-3 at 0.25 units/μl (bottom panel). The new peak (bottom panel) represented the expected mass of 1182 Da for the N-terminal product if cleaved after the aspartic acid as shown (the C-terminal product GRECGPH was not detected under these assay conditions). (E) Reverse phase HPLC of 1mM LP-Cterm (MW 2040 Da) following treatment with vehicle control (top panel), caspase-1 at 2.5 units/μl (middle panel), or caspase-3 at 0.25 units/μl (bottom panel). The caspase-1 and 3 cleavage products identified by mass spectrometry are indicated in the middle and lower panels. The new peaks generated represented the expected masses of 1127 Da (10 minute peak) and 931 Da (12 minute peak) (F) Depiction of full length LANA and the five different forms (cp1-cp5) of BCBL-1 LANA expected to be generated following caspase cleavage at the 2 caspase cleavage sites identified from peptide analysis.

To determine if oxidative stress altered the production and/or processing of latent proteins of KSHV, we treated BCBL-1 cells with increasing doses of H_2_O_2_, an inducer of caspase-dependent apoptosis, and probed for LANA by western blot. In the absence of H_2_O_2_, full length LANA (LANA-fl) was detected running at approximately 165 kDa and was predominantly in nuclear fraction as expected (Fig 1A). Also in control cells and H_2_O_2_ treated cells there was a band below full length LANA (approx. 120 kDa) that was detected in both the nuclear and cytoplasmic fractions (Fig 1A) and may represent the form of LANA produced through an alternate start site as described previously [27]. Interestingly, treatment with H_2_O_2_ led to an increase in LANA but also an increase in at least three faster migrating forms of LANA (molecular weights less than 165 kDa and > 100 kDa) in the nuclear fraction. We hypothesized that these faster migrating forms might have resulted from cleavage of LANA by caspases that were induced upon H_2_O_2_ treatment.

To assess whether LANA might in fact have sites susceptible to cleavage by caspases, we used the online program Cascleave [28] to analyze the LANA amino acid sequence for potential caspase cleavage sites. Two sites were revealed within the N-terminal domain of LANA and one site within the C-terminal domain that had high probability scores (> 0.8) for caspase cleavage (Fig 1B). In addition, there were a number of predicted cleavage sites within the acidic repeat region; however cleavage at these sites would not be expected to generate the forms of LANA we detected by western blot following H_2_O_2_ treatment. Based on the amino acid sequence, the first two sites identified in the N-terminus are predicted to be cleaved by group II caspases (caspases-2, -3 and -7) while the C-terminal site is predicted to be cleaved by group I caspases (caspase-1, -4, -5 and -13). Three peptides representing these three predicted cleavage sites in LANA were synthesized and each was treated with caspase-1 or caspase-3 and analyzed for peptide cleavage using reverse phase high performance liquid chromatography/mass spectrometry (RP-HPLC/MS). Two of these peptides, designated as LP-Nterm and LP-Cterm (Fig 1C) were found to be susceptible to caspase cleavage. LP-Nterm was not cleaved by caspase-1 but was readily cleaved by caspase-3 at its predicted cleavage site based on analysis by RP-HPLC/mass spectrometry, which revealed the expected mass for the large product of cleavage, RRKHVADSVD (the shorter product was not retained on the C18 column and therefore was not detected) (Fig 1D). LP-Cterm was cleaved by both caspase-1 and caspase-3 and yielded products with masses expected for cleavage at the predicted cleavage site (SSSEDEMEVD and YPVVSTHE) (Fig 1E). The LANA products that could be generated by caspase cleavage at the identified sites include a small 6 kDa N-terminal fragment (cp1), the corresponding 120.5 kDa C-terminal truncated fragment (cp2), a 102.5 kDa N-terminal truncated fragment (cp3), the corresponding 24 kDa C-terminal fragment (cp4) and a 96.5 kDa form of LANA that is truncated at both the N- and C-terminus (cp5) (Fig 1F).

**Fig 2:**
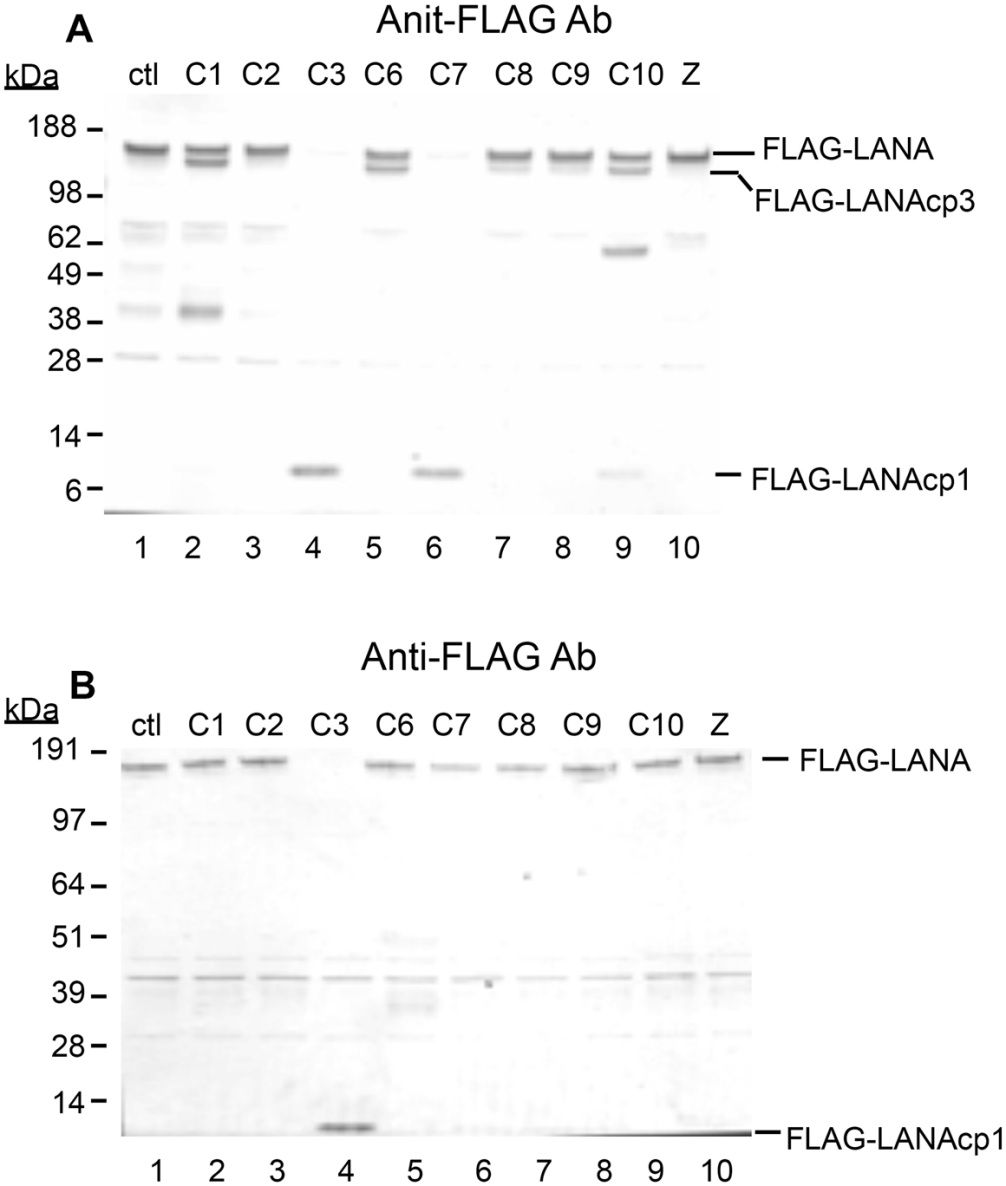
Caspases cleave FLAG-LANA *in vitro*. Caspase-1, 2, 3, 6, 7, 8, 9, 10 were tested for their ability to cleave LANA. HEK293T cells were transiently transfected with FLAG-LANA-pCMV, tagged with FLAG at the N-terminus as stated in Materials and Methods. Nuclear extracts containing LANA were harvested in the absence of protease inhibitors, treated with caspases for 16 hrs at 37°C and then analyzed by western blot. (A) FLAG-LANA treated with 0.15 units caspases μg^-1^ protein and probed with antibody to FLAG, anti-rabbit secondary antibody conjugated to alkaline phosphatase and visualized using stabilized Western Blue substrate (Promega). DMSO vehicle (lane 1) and 50 μM ZVAD (lane 10) were used as controls. FLAG-LANA and the primary cleavage products containing FLAG (FLAG-LANAcp1 and FLAG-LANAcp3) are indicated. Other products that increased in intensity to some of the caspases (caspase-1 and caspase-10) were not further investigated since these forms appeared to be present in the control extracts as well. (B) Same as Fig 2A except that a lower amount (0.07 units) of the caspases was used with an incubation time of only 4 hrs. Full-length FLAG-LANA (FLAG-LANA) and the primary caspase cleavage product (FLAG-LANAcp1) are indicated. FLAG-LANA (calculated molecular weight of 127 kDa) migrated as a band of approximately 160 kDa using the NuPage LDS gel system (InVitrogen). Molecular weight markers in kDa are indicated to the left of each blot. The molecular weight of FLAG is 1 kDa.

To determine if these were in fact bona fide caspase cleavage sites, we treated nuclear extracts containing N-terminal tagged FLAG-LANA with a number of different caspases. Eight caspases were tested for their ability to cleave FLAG-LANA produced in transfected HEK293T cells. Nuclear extracts of LANA incubated in the absence of caspases overnight for 16 hrs retained a prominent band detected with the antibody to the FLAG tag at approximately 160 kDa, indicative of full-length LANA (Fig 2A, lane 1). In addition, there were several less intense bands migrating further down on the blot. Some of these bands appeared to be generated by endogenous caspases in the extracts, since including the caspase inhibitor Z-VAD-FMK (ZVAD) in the absence of exogenous caspases (Fig 2A, lane 10) decreased or eliminated most of them (Fig 2A; compare Lane 1 with lane 10). There was some processing of FLAG-LANA detected by all the caspases, except caspase-2, as evidenced by a decrease in full length FLAG-LANA and/or the production of new FLAG-containing products migrating either just below full length LANA or migrating as a low molecular weight product near the bottom of the blot (Fig 2, lanes 2–9). The low molecular weight form of FLAG-LANA was consistent with cleavage occurring at the N-terminal site identified by peptide analysis and is denoted as FLAG-LANAcp1 (Fig 2A). The form of LANA migrating just below full length LANA was consistent with cleavage occurring at the C-terminal site identified by peptide analysis and is denoted as FLAG-LANAcp3 (Fig 2A). In addition to these forms of LANA, some of the bands detected in the absence of added caspase (Lane 1) increased in intensity following addition of caspase-1 and -10, thus indicating the potential for additional caspase cleavage sites in LANA. Caspases-3 and 7, which are known to have similar substrate preferences, were particularly effective at cleaving FLAG-LANA as evidenced by a nearly compete loss of native FLAG-LANA that coincided with the generation of FLAG-LANAcp1 at approximately 6kD; this suggested that both caspase-3 and caspase-7 cleave LANA at the same site in the N-terminus (Fig 2A, lanes 4 and 6). Caspase-10 also generated a small amount of FLAG-LANAcp1 (Fig 2A, lane 9) while caspase-1, 6, and 10 generated a prominent cleavage product detected with the FLAG antibody migrating at approximately 110–120 kDa (denoted as FLAG-LANAcp3) (Fig 2A, lanes 2, 5, and 9), indicating that cleavage of LANA by these caspases was occurring at the C-terminus (as indicated by the retention of the FLAG tag). Also, when probed with an antibody to LANA instead of FLAG, the FLAG-LANA samples treated with caspase-3 and caspase-7 contained forms of LANA corresponding to those expected following cleavage at the N- and N-and C-terminal sites (S1 Fig). FLAG-LANA was also exposed to caspases at one-half the enzyme concentration and for only a 4 hr incubation period (Fig 2B). This revealed that FLAG-LANA was particularly susceptible to cleavage by caspase-3 under the conditions used (Fig 2B, lane 4). While there was also some decrease in intensity of FLAG-LANA following treatment with caspase-7 and caspase-8, only caspase-3 efficiently eliminated native FLAG-LANA under these conditions and generated the N-terminal fragment, FLAG-LANAcp1 (Fig 2B, lane 4).

**Fig 3:**
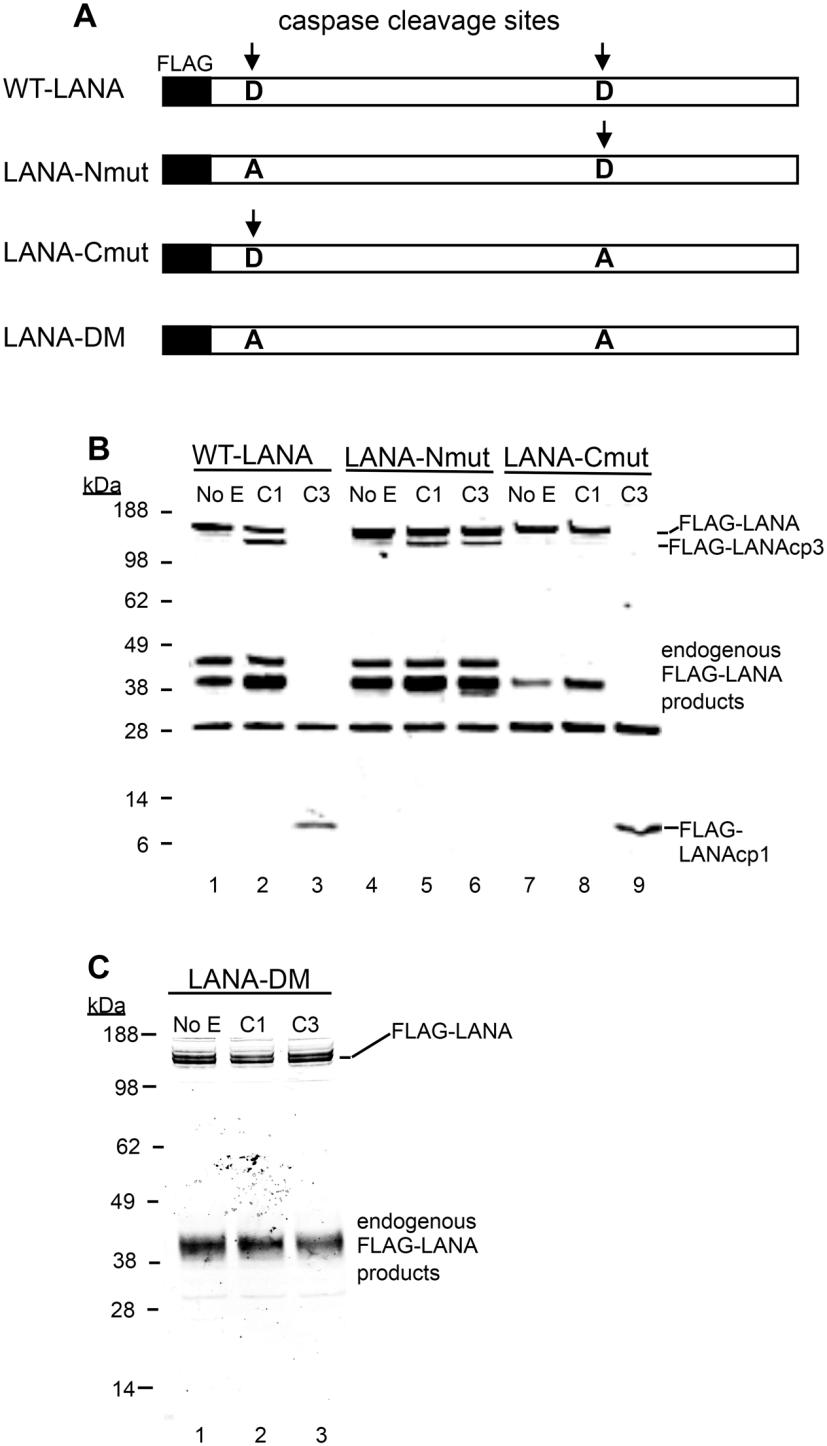
Mutation of putative LANA caspase cleavage sites at the N- and C-termini of LANA prevent caspase cleavage of LANA. (A) A schematic diagram showing the FLAG-WT-LANA (WT-LANA) and the location of two caspase cleavage sites identified through sequence and peptide analysis. FLAG is present at the N-terminus of LANA in these constructs and the aspartate cleavage sites susceptible to caspase cleavage are indicated with an arrow. Three mutant forms of FLAG LANA were prepared. FLAG-LANA-Nmut (LANA-Nmut) contains an Asp to Ala (A->D) mutation at the N-terminal site, FLAG-LANA-Cmut (LANA-Cmut) contains an A->D mutation at the C-terminal site and FLAG-LANA-DM (LANA-DM) has both sites mutated from A->D. (B) HEK293T cells were transiently transfected with expression vectors producing FLAG-tagged forms of WT-LANA, LANA-Nmut or LANA-Cmut (labeled as in 3A). Following transfection, nuclear extracts were harvested and treated for 16 hrs at 37°C with PBS (control), caspase-1 (C1) or caspase-3 (C3) (0.15 units caspases/μg protein). FLAG-LANA and cleavage products containing FLAG were detected by western blot with an antibody to FLAG as in Fig 2. The FLAG-LANA cleavage products are indicated as FLAG-LANAcp3 (cleavage occurring at the C-terminus) or FLAG-LANAcp1 (cleavage occurring at the N-terminus). While the identity of these products is not defined, the labeling scheme is based on that diagrammed in Fig 1F to help visualize the products. Note that several endogenous FLAG-LANA products were seen in control samples generated by endogenous enzymes in the extracts. (C) FLAG-LANA-DM (LANA-DM) (lanes 1–3) was treated with caspase-1 or -3 (0.15 units caspases/μg protein) and then analyzed by western blot using anti-FLAG antibody. The FLAG-LANA cleavage products again are indicated. Data shown are representative of 3 independent experiments in (B) and two independent experiments in (C). Molecular weight markers in kDa are indicated to the left of each blot.

To determine if LANA was in fact cleaved at the two sites identified by sequence and peptide analysis, FLAG-LANA expression plasmids containing Asp (D) to Ala (A) mutations at the identified cleavage sites were prepared (Fig 3A) and cleavage by capsases-1 and -3 was analyzed using anti-FLAG antibodies. Plasmids denoted as LANA-Nmut and LANA-Cmut were created which contained a mutation at the N-terminal cleavage site or the C-terminal cleavage site, respectively, and these were compared to WT-LANA in the cleavage assay. As seen before, Caspase-1 cleaved wild type FLAG-LANA to generate a large FLAG-tagged fragment migrating on western blot just below full length FLAG-tagged LANA (designated FLAG-LANAcp3), while treatment with caspase-3 eliminated detection of full length LANA and generated a small 6 kDa fragment (designated FLAG-LANAcp1) (Fig 3B, compare lanes 1, 2 and 3). Caspase-1 was still able to cleave LANA-Nmut as evidenced by the continued production of FLAG-LANAcp3 indicating that cleavage occurred at the C-terminal site (Fig 3A, compare lanes 2 and 5). By contrast, the LANA-Nmut was not cleaved at the N-terminus by caspase-3, as it no longer generated FLAG-LANAcp1. This confirmed that caspase-3 cleaves at the N-terminal site of WT FLAG-LANA (Fig 3B, compare lanes 3 and 6). At the same time, the LANA-Nmut was cleaved by caspase-3 at the C-terminus to generate FLAG-LANAcp3 indicating that the C-terminal cleavage site was susceptible to cleavage by both caspase-1 and -3 (Fig 3B, lanes 4,5 and 6); this is consistent with the enzyme susceptibility of the peptides mimicking these cleavage sites (Fig 1D and 1E) and suggests that caspase-3 preferentially cleaves at the N-terminus even though it can cleave at both sites. Mutation of the C-terminal cleavage site (LANA-Cmut) eliminated the caspase-1-induced generation of FLAG-LANA-cp3, thus confirming that this cleavage site is susceptible to caspase-1 (Fig 3B, compare lanes 2 and 8). LANA-Cmut was still cleaved by caspase-3 at the N-terminus as expected and produced FLAG-LANAcp1 (Fig 3B, lane 9). We also generated a double mutant form of FLAG- LANA that had both cleavage sites mutated (denoted LANA-DM) to assess if LANA would no longer be susceptible to either caspase within the same LANA construct. FLAG-tagged LANA with mutations at both cleavage sites was not cleaved by either capsase-1 or -3 (Fig 3C, lanes 1–3). These data confirm that LANA contains a specific caspase cleavage site at the N-terminus that is susceptible to capsase-3 and a C-terminal site that is susceptible to both caspase-1 and -3.

### H_2_O_2_ induces changes in LANA in KSHV-infected cells that are blocked by caspase inhibition

**Fig 4:**
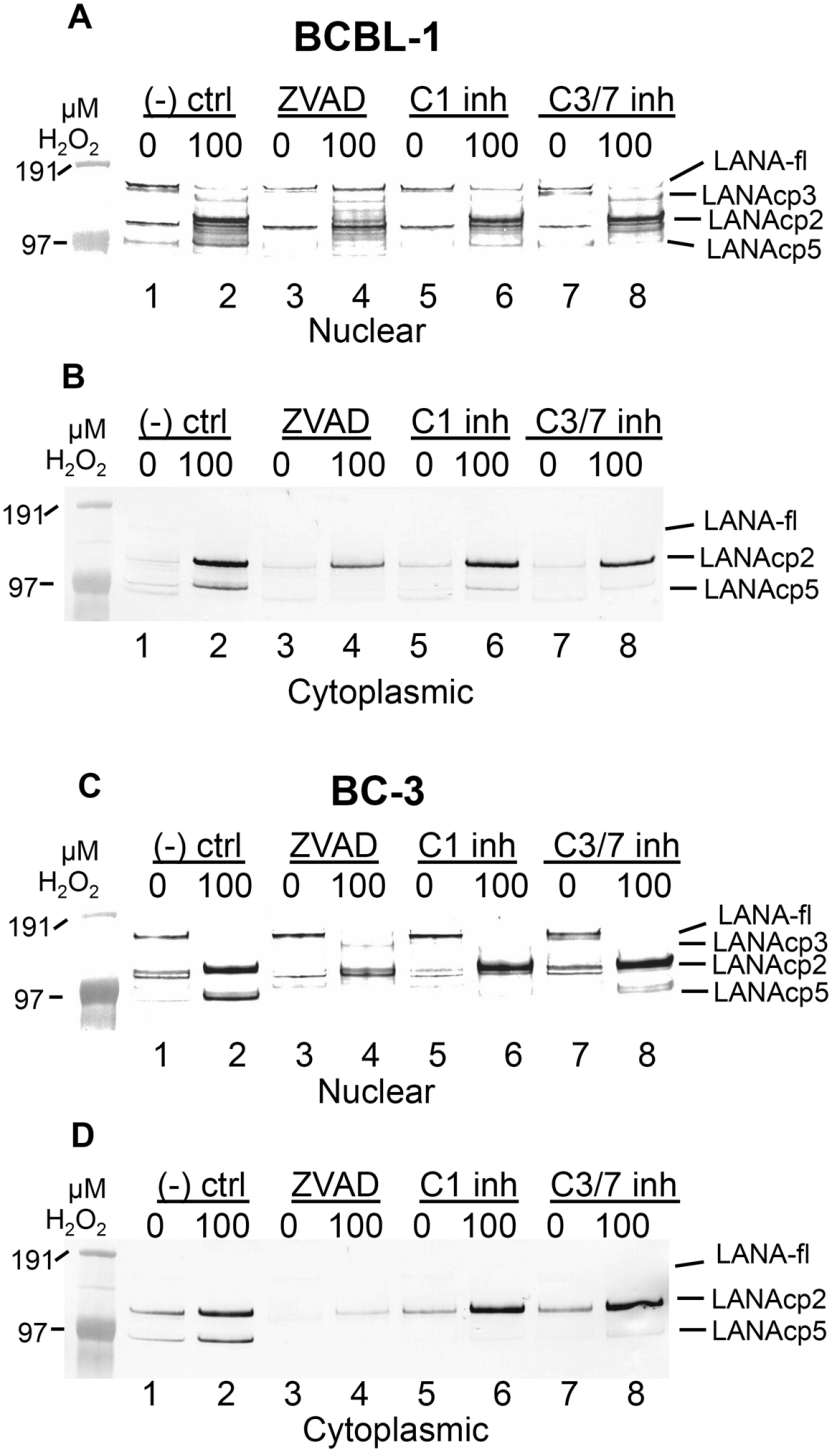
Caspase inhibitors block oxidative stress-induced changes in LANA. BCBL-1 (A, B) or BC-3 (C, D) cells were treated with PBS vehicle control or 100 μM H_2_O_2_ in the presence of 50 μM Z-FA-FMK (- ctrl), a negative control peptide (lanes 1 and 2), ZVAD-FMK (ZVAD), a pan-caspase inhibitor (lanes 3 and 4), a specific inhibitor of caspase-1 (C1 inh) (lanes 5 and 6) or a specific inhibitor of caspase-3/7 (C3/7 inh) (lanes 7 and 8). Nuclear (A, C) and cytoplasmic (B, D) extracts were harvested and LANA expression was analyzed by western blot and probed with antibody to FLAG, anti-rabbit secondary antibody conjugated to alkaline phosphatase and visualized using stabilized Western Blue substrate (Promega). Results are representative of 3 separate experiments for controls and ZVAD and 2 separate experiments for the C1 and C3/7 inhibitors. Molecular weight markers are shown to the left. To the right of the blots the different forms of LANA are indicated and include the location for full length LANA (LANA-fl) and three forms of LANA migrating below full length LANA with presumptive designations as LANAcp3, LANAcp2 and LANAcp5 based on their mobility.

The identification of bona fide caspase cleavage sites in LANA suggested that these sites might be cleaved under conditions where caspases are activated in KSHV-infected cells. In the initial experiments described previously (see Fig 1A) in which treatment of BCBL-1 cells with H_2_O_2_ led to faster migrating forms of LANA, it was noteworthy that the bands migrating below full length LANA had estimated molecular weights consistent with the approximate sizes expected if caspase cleavage of LANA was occurring at either or both of the sites identified in the N- and C-terminus of FLAG-LANA. To further explore this hypothesis, we tested the effect of caspase inhibitors on the H_2_O_2_-induced changes in LANA in both BCBL-1 and BC-3 cells in cytoplasmic and nuclear extracts. Cells were pretreated with a negative control peptide (Z-FA-FMK), the pan-caspase inhibitor ZVAD, or specific inhibitors of either caspase-1 or caspase-3/7, and then exposed to H_2_O_2_. H_2_O_2_ led to a loss of full length LANA and an increase in at least three faster migrating forms of LANA, tentatively designated as LANAcp3, LANAcp2 and LANAcp5 based on their hypothesized positions within LANA as depicted in Fig 1F (Fig 4A, 4B, 4C and 4D, compare lanes 1 and 2). In nuclear extracts of BCBL-1 cells ZVAD treatment eliminated the production of LANAcp5, decreased the levels of LANAcp2 (upper band of the doublet), and increased the levels of full length LANA (LANA-fl) (Fig 4A compare lanes 2 and 4). Similar results were obtained for nuclear extracts of BC-3 cells except that full length LANA was not noticeably restored by ZVAD (Fig 4C compare lanes 2 and 4). Overall, these results indicated that full length LANA was being processed to faster migrating forms by cellular caspases in PEL cells. Changes in LANAcp3 differed between the cell lines, probably reflecting a balance between production and further caspase cleavage to lower MW forms. Interestingly, in BC-3 cells ZVAD even decreased the levels of LANAcp2 and modestly increased the levels of full length LANA in mock treated cells without H_2_O_2_ (compare lanes 1 and 3 in Fig 4C) suggesting that a low level of caspase cleavage is takes place in BC-3 cells even in the absence of H_2_O_2_ treatment. Specific inhibitors of capsase-1 and caspase 3/7 were both able to decrease the production of the LANAcp5 band induced by H_2_O_2_ in both PEL lines but had little or no effect on the LANAcp2 band (Fig 4A and 4C compare lanes 2, 6 and 8). In BCBL-1 cells the caspase-1 inhibitor appeared to eliminate the LANAcp3 band detected just under LANAfl while the caspase-3/7 inhibitor did not (compare lanes 2, 6 and 8 in Fig 4A).

In cytoplasmic extracts, the inhibition of H_2_O_2_-induced changes in LANA by caspase inhibitors was quite obvious. LANAcp2 and LANAcp5 were prominent in cytoplasmic extracts of H_2_O_2_-treated samples from BCBL-1 cells and BC-3 cells while LANAp1 and full length LANA were essentially undetectable (Fig 4B and 4D lane 2). Treatment with ZVAD decreased the production of LANAcp2 and LANAcp5 substantially in cytoplasmic extracts (Fig 4B and 4D; compare lanes 2 and 4). While the caspase-1 and caspase-3/7 inhibitors prevented the accumulation of LANAcp5 in cytoplasmic extracts of both cell lines, they had only a limited effect on LANAcp2 in cytoplasmic extracts of BCBL-1 cells and no effect on LANAcp2 in cytoplasmic extracts of BC-3 cells. This suggests that a caspase besides caspase-1 or -3/7 may contribute to the production of LANAcp2, or that the levels of LANAcp2 reflect a balance between production and further cleavage. Nevertheless, these data provide strong evidence that LANA undergoes low levels of cleavage in PEL cells by caspases and this increases substantially during H_2_O_2_-induced apoptosis in these cells. Based on the processing sites we identified in FLAG-LANA, this data suggests that LANAcp2 may represent LANA cleaved at the N-terminal site, LANAcp3 as LANA cleaved at the C-terminal site and LANAcp5 representing LANA cleaved at both sites as diagrammed in Fig 1F.

The generation of LANA bands migrating below full length LANA were not only observed following treatment with H_2_O_2_ in BCBL-1 cells and BC-3 cells but also with Bay-11, an inducer of oxidative stress in PEL lines [24], and with etoposide, an inducer of apoptosis (S2A and S2F Fig). Interestingly, these treatments not only led to processing of LANA, but also appeared to increase the overall levels of full length LANA as well, especially at the lower treatment doses (S2A Fig).

### LANA can inhibit caspase activity by acting as a substrate decoy

**Fig 5:**
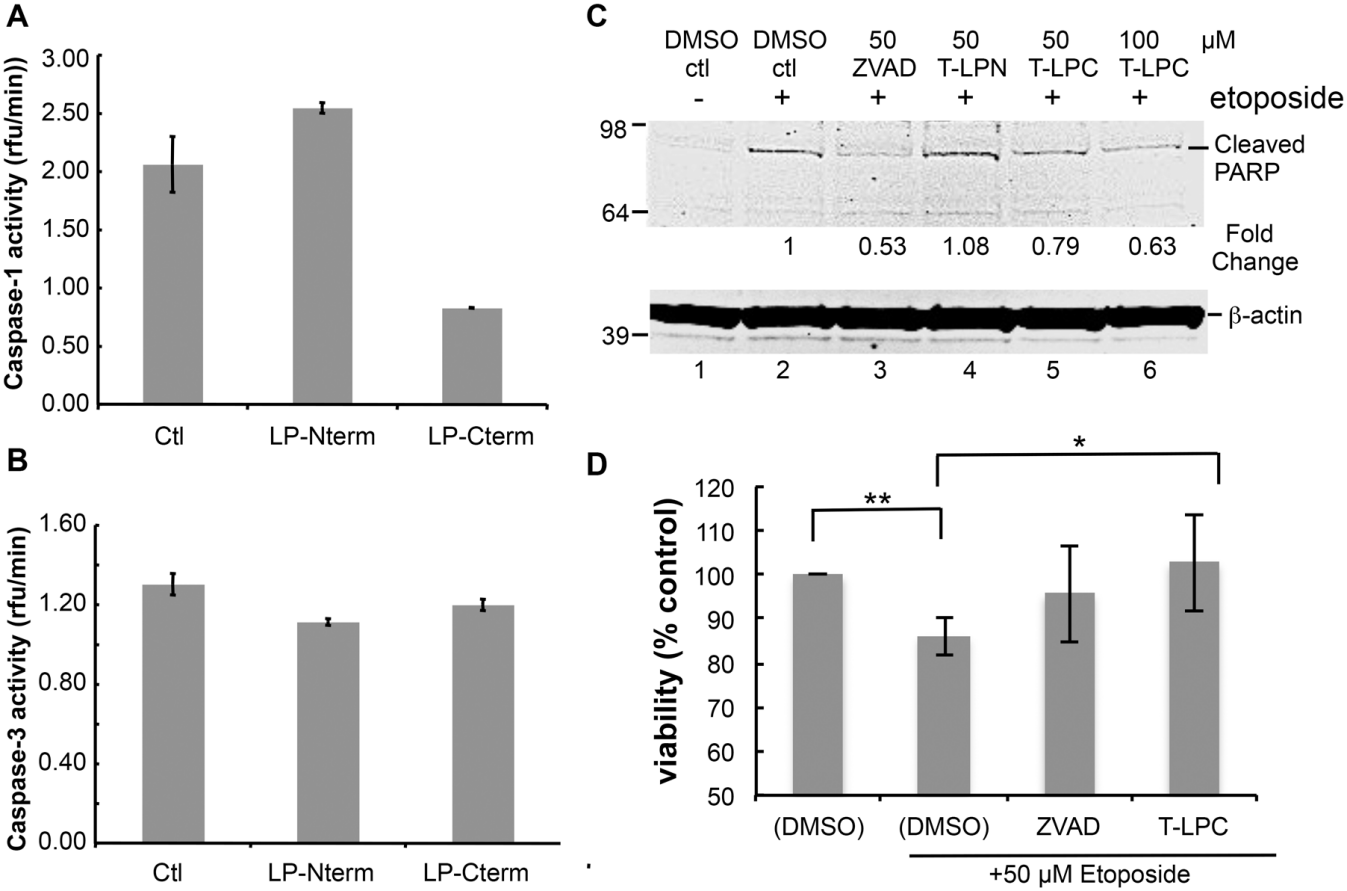
Peptides containing LANA caspase cleavage sites inhibit caspase activity, decrease PARP cleavage and increase cell viability. Caspase activity assays were performed as described in Materials and Methods. Peptides containing the N-terminal caspase cleavage site of LANA (LP-Nterm) or the C-terminal caspase cleavage site of LANA (LP-Cterm) were dissolved in PBS and tested as potential caspase inhibitors at a concentration equal to the commercial caspase-1 substrate (Ac-YEVD-pNa, 200 μM, Sigma) or caspase-3 substrate (Ac-DEVD-pNa, 200 μM, Sigma). PBS was used as the negative control. (A) Effect of LP-Nterm and LP-Cterm on caspase-1 activity. (B) Effect of LP-Nterm and LP-Cterm on caspase-3 activity. In both A and B, the linear caspase activity rate (rfu/min) is plotted with the assay run in triplicate in two separate experiments with one representative experiment shown. Note: The increase in activity of caspase-1 by the LP-Nterm may be due to the presence of a cysteine in this peptide which could protect loss of activity of the enzyme due to oxidation over time. (C) HEP3B cells were pre-treated with DMSO, ZVAD, Tat-LP-Nterm (T-LPN) or Tat-LP-Cterm (T-LPC) peptide for two hrs and then exposed to DMSO alone (control) or etoposide for 16 hrs at 50 μm to induce apoptosis and PARP cleavage. Cell lysates were analyzed by western blot for cleaved PARP and β-actin (loading control). The percent inhibition of PARP cleavage as determined using the LiCor system is indicated below the blot. The immunoblot shown is a representative of 3 different experiments with Tat-LP-Nterm and Tat-LP-Cterm at 50 μM (Tat-LP-Cterm at 100 μM done once). (D) Cell viability following pretreatment with DMSO, ZVAD or T-LPC followed by DMSO or etoposide treatment. Cells were pretreated with DMSO (control), Tat-LP-Cterm or ZVAD (50 μM) for two hrs followed by etoposide treatment at 50 μM. To assess cell viability total cellular protein obtained from the adherent cells following 3 PBS washes was determined by BCA assay. The results shown are the average of 4 independent experiments. ** P< 0.01, * P< 0.05, for two tailed Student’s t-test.

Many viruses encode proteins that either function to prevent apoptosis or otherwise utilize caspase cleavage to their advantage (for review see [5]). Therefore, we considered the possibility that these LANA cleavage sites may function as substrate decoys for caspases and by this mechanism delay or prevent various caspase-mediated events. To determine if LANA cleavage sites could act as decoys for caspase-1 and -3, we first tested the LP-Nterm and LP-Cterm peptides containing the LANA cleavage sites for inhibition of caspase-1 and caspase-3 activity *in vitro*. While caspase-1 activity was slightly increased in the presence of LP-Nterm, activity was inhibited in the presence of LP-Cterm by approximately 60% when tested at a concentration equal to that of the assay substrate (Ac-YEVD-pNa) (Fig 5A). In addition, caspase-3 activity was minimally inhibited by both LP-Nterm and LP-Cterm when tested under the same conditions against the Ac-DEVD-pNa assay substrate (15% and 8% decrease, respectively) (Fig 5B). These data indicated that the LANA cleavage sites, especially the C-terminal site (LP-Cterm), might be able to delay caspase-mediated events by decreasing cleavage of their intended cellular targets. To test this hypothesis, Tat-LP-Nterm and Tat-LP-Cterm (cell-permeable forms of these LANA peptides prepared with a 9 amino acid human immunodeficiency virus Tat leader sequence to facilitate cell entry [29]) were used in a cellular assay in which poly ADP ribose polymerase (PARP) is cleaved by caspases following etoposide treatment of HEP3B cells. ZVAD was used as a control. The levels of cleaved PARP can be used to assess cellular caspase activity during apoptosis [30,31]. There was virtually no detectable cleaved PARP in cells treated with vehicle alone but cleaved PARP appeared following treatment with etoposide (Fig 5C, lanes 1 and 2). The pan-caspase inhibitor ZVAD effectively inhibited the production of cleaved PARP following etoposide treatment (Fig 5C, lane 3). ZVAD and Tat-LP-Cterm, but not Tat-LP-Nterm, consistently decreased the amount of cleaved PARP induced by etoposide (ZVAD decreased PARP cleavage by 47% while LP-Cterm decreased PARP cleavage by 21% at 50 μM and 37% inhibition by LP-Nterm at 100 μM based on Image Studio analysis) (Fig 5C, lanes 3–6). Also, the decrease in PARP cleavage coincided with protection from etoposide-induced toxicity by LP-Cterm, consistent with an inhibition or delay in apoptosis (Fig 5D).

**Fig 6:**
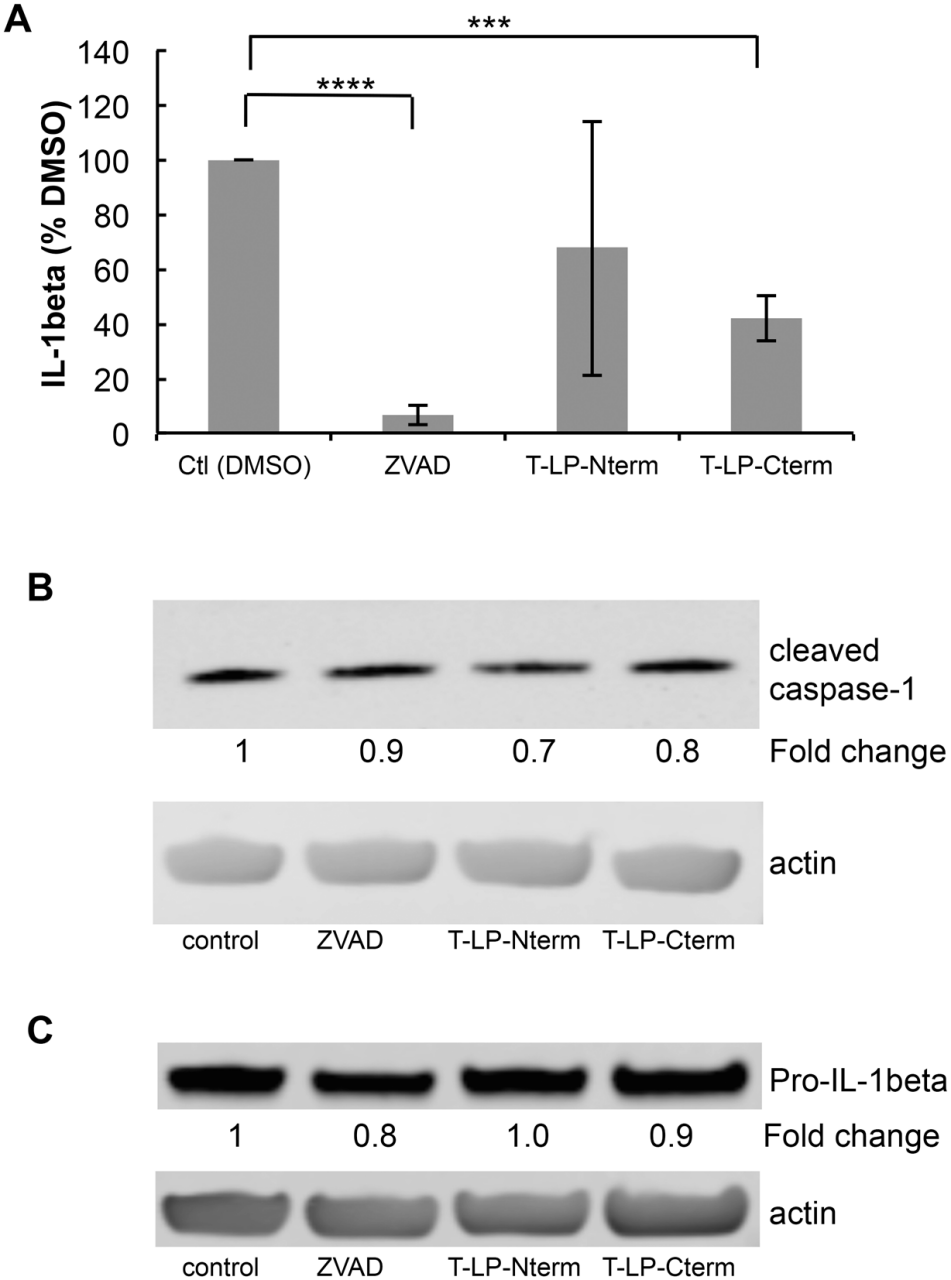
Peptides containing LANA caspase cleavage sites inhibit IL-1β production. Cell permeable peptides with an N-terminal cellular HIV-1 Tat delivery sequence RKKRRQRRR containing the N-terminal caspase cleavage site of LANA (T-LP-Nterm) or the C-terminal caspase cleavage site of LANA (T-LP-Cterm) were tested as potential inhibitors of IL-1β production following treatment of THP-1 cells with LPS. ZVAD was used as a positive caspase inhibitor control. (A) Effect of ZVAD (50 μM), T-LP-Nterm (50 μM) and T-LP-Cterm (50 μM) on IL-1β in the supernatant from THP-1-treated cells. Δata is from five separate experiments (except for T-LP-Nterm, 4 experiments). ***, *P*< 0.0005, **** *P*< 0.0001 for two tailed paired Student’s t-test. The average IL-1β λεωελ ϕορ untreated and LPS treated THP-1 cells was 276 pg/ml and 2344 pg/ml, respectively. (B) Immunoblot analysis for cleaved (mature) caspase-1 and actin as a loading control. The fold change in cleaved caspase expression normalized to β-actin is shown just below the immunoblot using the LiCor system. (C) Immunoblot analysis for Pro-IL-1β and actin as a loading control. The fold change in Pro-IL-1β expression normalized to β-actin is shown just below the immunoblot using the LiCor system.

Recent studies have shown that KSHV-infected cells, including PEL cells, manifest continuous activation of the inflammasome, a host response to infection [6]. Induction of the inflammasome leads to the production of active (cleaved) capsase-1 from pro-capase-1, in turn resulting in the production and secretion of interleukin-1beta (IL-1β) and interleukin-18 (IL-18). These cytokines are part of the innate immune response. We assessed whether the caspase cleavage sites in LANA might inhibit the production of IL-1β by blocking caspase-1 activity. LPS induction of the inflammasome in THP-1 monocytes was used as a model system to investigate cytokine levels in the absence and presence of Tat-LP-Nterm or Tat-LP-Cterm. ZVAD was utilized as a positive control for caspase inhibition. Matured THP-1 cells were pretreated for 2 hrs with Tat-LP-Nterm, Tat-LP-Cterm or ZVAD, induced with LPS, cultured for 20 hrs, and IL-1β in the supernatant collected and was assayed by ELISA. In the absence of inhibitors, LPS induced almost a 10- fold increase (average of 276 pg/ml without LPS and 2344 pg/ml with LPS) in IL-1β The increase induced by LPS was inhibited by more than 90% in the presence of ZVAD (50 μM) (Fig 6A). Tat-LP-Nterm (50 μM) did not consistently alter levels of IL-1β induced by LPS (Fig 6A). However, Tat-LP-Cterm (50 μM) consistently inhibited the IL-1β production in the presence of LPS (average 57% inhibition) (Fig 6A). Although the levels of IL-1β deceased in response to ZVAD and Tat-LP-Cterm, these treatments did not lead to substantial changes in levels of cleaved caspase-1 in the presence of LPS as assessed by western blot, indicating these treatments likely function to inhibit caspase activity rather than by decreasing caspase protein levels (Fig 6B). Also, the level of Pro-IL-1β from which IL-1β is derived was not substantially changed by these treatments, suggesting that the decrease in IL-1β was likely due to the inhibition of caspase-1 rather than decreasing the levels of the Pro-IL-1β substrate (Fig 6C).

**Fig 7:**
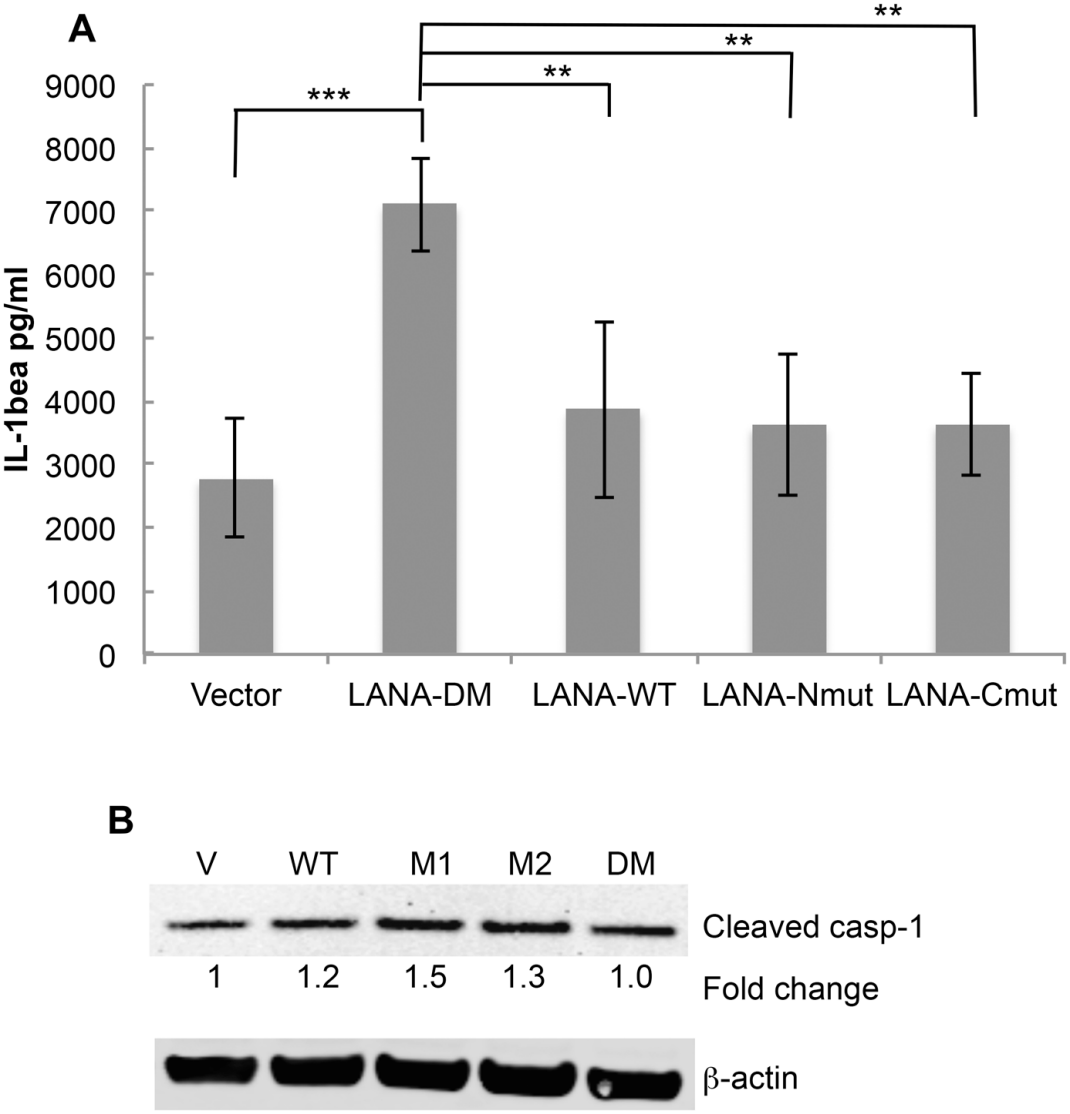
Mutation of both LANA caspase cleavage sites leads to increased IL-1β production. THP-1 cells were matured overnight by treatment with TPA and then transiently transfected with FLAG-tagged forms of LANA-DM, WT-LANA, LANA-Nmut, or LANA-Cmut. The next day cells were treated with LPS and the level of IL-1β in the supernatants measured 20 hrs later. Cell extracts were also made for protein analysis and immunoblots. (A) IL-1β levels as determined by ELISA following transfection with the LANA plasmid constructs and treatment with LPS. The average level of IL-1β detected following transfection with vector alone (no LPS) was 2131 pg/ml (S3 Fig). (B) Immunoblot for cleaved caspase-1 and actin showing the relative levels of active casapse-1 compared to vector alone for the LPS-treated cells using the LiCor system. Data shown in (A) are the average +/- the standard deviation from 4 independent experiments. ** *P*< 0.01, *** P<0.005 for two-tailed Student’s t-test. Note that the average values for the single mutants were not statistically significantly different (p>0.05) when compared to wild type.

To determine if the cleavage sites in the context of full length LANA functioned to blunt IL-1β προδυχτον, we transfected THP-1 cells with the plasmids encoding either vector control, wild type LANA, LANA-Nmut, LANA-Cmut or LANA-DM. We compared the levels of IL-1β following transfection with the different LANA constructs following LPS treatment. LPS-treated cells transfected with LANA-DM secreted substantially more IL-1β than the vector control, possibly reflecting additional activation of the inflammasome by the transfected LANA DNA or protein (Fig 7A) like that seen with other viral DNA and protein constructs [32–34]. However, compared to LANA-DM, the wild-type (LANA-WT) had a 45% decrease in the production of IL-1β as did either of the single LANA mutants (*P*<0.01 in each case) (Fig 7A). These results indicate that the presence of either cleavage site may function to blunt production of IL-1β. We also evaluated the levels of active caspase-1 following transfection. This data indicated that the increase of IL- β seen with the LANA-DM construct could not be attributed to and increase in active caspases-1 as it had similar levels as the vector control and even lower levels than the other LANA constructs (Fig 7B). Even in the absence of LPS treatment, the presence of either or both of the wild type caspase cleavage sites led to an almost 2-fold decrease in the levels of IL-1β detected in the supernatant as compared to the double mutant (S3A and S3B Fig). Taken together, these results provide evidence that LANA can inhibit caspase-1 cleavage of the IL-1β precursor and suggest that both caspase cleavage sites may contribute to this inhibition. Of note, IL-18 levels were also measured in the supernatant from the LANA-transfected cells in these experiments. However, unlike IL-1β, the production of IL-18 was not inhibited by cell-permeable LANA peptides and did not differ between the various LANA constructs (S4 Fig).

## Discussion

Using sequence, peptide and mutational analysis, we identified two caspase cleavage sites within KSHV LANA: one located in the N-terminal region and the other in the C-terminal region. These two sites could be cleaved by caspase-1 and/or -3, and mutation of the critical aspartate cleavage sites prevented LANA cleavage. We also demonstrated that when KSHV-infected cells were exposed to agents that induce caspase activation and apoptosis, LANA underwent caspase-dependent changes leading to the generation of forms of LANA with lower apparent molecular weights. These changes in LANA could be blocked, in part, by the presence of the global caspase inhibitor ZVAD, indicating that LANA is susceptible to caspase cleavage in infected cells. Using peptides mimicking the cleavage sites, as well as wild type and mutant forms of LANA expression vectors, we provide evidence that these sites can function to blunt cellular defense responses including apoptosis and the inflammasome.

In BC-3 and BCBL-1 cells exposed to H_2_O_2_, there was an increase in three faster migrating bands that reacted to LANA antibodies. Although inhibitors of caspase-1 and caspase-3/7 could decrease some of the processed forms of LANA, ZVAD was clearly more effective, suggesting that other caspases may be involved in LANA cleavage as well. Taken together, these results suggest that caspase cleavage of LANA accounts at least in part for the multiple bands observed when western blots of KSHV-infected cells are probed with anti-LANA antibodies. Among the new forms of LANA, LANAcp3 was detected in the nuclear extracts while LANAcp3 and LANAp5 were present in both the nuclear and cytoplasmic extracts. This data suggests that cleavage of LANA at these sites affects the cellular distribution of LANA. Full length LANA is primarily a nuclear protein [35], and two nuclear localization sequences (NLS) of LANA are located in the N-terminus [36] consistent with the detection of LANAcp3 exclusively in the nucleus (which also retains these NLS). Caspase cleavage at the N-terminal cleavage site would remove the NLS and as a result N-terminal truncated forms of LANA would tend to accumulate in the cytoplasm. Caspase-1 cleavage of LANA also generates a 214 amino acid C-terminal fragment. It is interesting to note that others have shown that this portion of LANA self-associates and enters the nucleus [37]. This suggests that the C-terminal fragment generated by caspase cleavage could enter the nucleus and function in ways differently than full length LANA. Toptan et al. have recently reported another mechanism by which a form of KSHV LANA that accumulates in the cytoplasm can be produced: by translation of a truncated form of LANA [27]. The existence of two complementary mechanisms for producing LANA that can migrate to the cytoplasm suggests that these forms may play important roles for the virus, and the results here suggest that one benefit may be to inhibit caspases and apoptotic and inflammatory events that occur in the cytoplasm.

The N-terminal site in FLAG-LANA was susceptible to cleavage by caspase-3, while the C-terminal site was susceptible to cleavage by caspase-1 and caspase-3. This finding, combined with the observation that specific inhibitors of caspase-1 and caspases-3/7 also inhibited the changes in LANA in infected cells, provides strong evidence that caspases cleave LANA in KSHV-infected cells undergoing oxidative stress-induced apoptosis. Caspases-7 and -10 also were able to cleave FLAG-LANA at the same N-terminal site as caspase-3, although they did not appear as active under the same conditions. Also, caspases-6 and -10 cleaved LANA at the same C-terminal site as caspase-1. Based on our studies with FLAG-LANA we can tentatively assign LANAcp3 (Figs 1 and 4) as a large fragment of LANA lacking the N-terminal 53 amino acids due to caspase cleavage. LANAcp2 is consistent with a form of LANA lacking the C-terminal 212 amino acid fragment while LANAcp5 may be LANA lacking both the N and C-terminal fragments. Clearly other forms of LANA may also be present and our identification of these products in infected cells is preliminary, in part, because a heterogeneous population of LANA proteins is produced in PEL lines even before cleavage due to transcriptional variations [27] and posttranslational modifications [38–40].

We explored why KSHV may have evolved to have sites in LANA that were cleaved by caspases. We hypothesized that one benefit to KSHV would be for these sites to serve to inhibit or attenuate cellular defense responses. One of the defenses against virus infection is apoptosis; by this mechanism, virus-infected cells self-destruct, thereby preventing virus replication and spread [1,2,5]. Inhibition of apoptosis is especially important for latent viruses, such as KSHV, and several mechanisms by which KSHV can inhibit apoptosis have previously been described [13,14,41–43]. There are also numerous examples of viruses evolving mechanisms to directly inhibit caspases [5]. The p35 protein of baculovirus directly inhibits several caspases and the cowpox virus protein, CrmA, directly inhibits caspase-1 [5]. Here we propose that KSHV may inhibit apoptosis by using LANA as a substrate decoy to blunt caspase activity. We found that peptides spanning the LANA cleavage sites acted as competitive inhibitors in standard caspase activity assays, thus providing evidence that LANA, which is abundantly produced in KSHV-infected cells, may prevent or delay caspase-mediated events. In particular, the C-terminal peptide of LANA, which is susceptible to cleavage by both caspase-1 and -3, was able to decrease the extent of PARP cleavage in HEP3B cells treated with etoposide and this coincided with an increase in cell viability. Interestingly, it has been reported that KSHV infection confers a survival advantage to endothelial cells in the presence of etoposide [44]; the results here suggest that LANA caspase cleavage sites may, at least in part, play a role in this process.

We also explored the possible role of LANA’s caspase cleavage sites in blocking the production of IL-1β that occurs due inflammasome-mediated activation of caspase-1 [6,45,46]. Previous studies have shown that certain viruses encode proteins that inhibit caspase-1 activity and therefore the production of inflammatory cytokines. For example, CrmA, a product of cowpox virus, was shown to inhibit IL-1β and IL-18 production. Also, studies on KSHV-infected PEL cells [6] and endothelial cells [47] show that the inflammasome is activated in latently infected cells and therefore mechanisms for blunting the downstream events of inflammasome activation during latency would be advantageous. Although other studies have described ORF 63 of KSHV as a protein that blunts the inflammasome, this lytic protein would primarily function during the lytic cycle and is generally not produced in latency [17,48]. In this study, we found that a cell-permeable peptide of the C-terminal cleavage site in LANA effectively decreased the levels of IL-1β in the supernatant of cells although the N-terminal site had inconsistent effects. In addition, transfection experiments showed that compared to wild-type LANA, LANA with mutations of the two caspase cleavage sites led to a significant increase in IL-1β production, suggesting that these sites help to blunt the level of IL-1β production in latently infected cells. To our surprise, however, we did not see similar effects on IL-18 production in cells treated with the peptides or transfected with the LANA plasmids, even though IL-18 is usually processed through the caspase-1 pathway. It is possible that the affinity of caspase-1 for the peptides and LANA is less than that for pro-IL-18 or that production of IL-18 involves other proteolytic pathways. The cleavage sites of IL-1β and IL-18 do indeed differ (YVHD and LESD, respectively) and this may explain the anomaly. Also, while caspase cleavage inhibition may explain the effects on IL-1β, it remains possible that other mechanisms are involved in production of IL-1β and/or IL-18.

While our data support a role for LANA in blunting cellular defense responses, the caspase cleavage sites could also function in other ways. LANA is a multifunctional protein essential for maintaining latency and KSHV persistence. Many functional domains of LANA are in the N- and C-terminal regions, separated by a highly acidic repeat region. Consequently, the caspase cleavage of LANA may confer a gain or loss of a number of different functions and these would have to be addressed in future studies. Some cleavage products may have unanticipated functions. Also, based on what is known about LANA, we can hypothesize about some possible additional roles for these sites or products derived from LANA cleavage. For example, since the binding site for RTA is in the C-terminal domain of LANA [49] it is possible that caspase cleavage at the C-terminus could interfere with RTA binding to LANA, thus resulting in activation of the lytic cycle. This is plausible given that oxidative stress has been shown to reactivate KSHV from latency [24,25]. Also, cleavage of LANA at the N-terminal domain may alter the ability of LANA to bind HIF-1α which has been shown to be important in RTA activation [50]. In a similar manner, it has been suggested that caspase-3 may induce KSHV lytic replication through a mechanism that does not involve RTA [51], and it is conceivable that a LANA caspase cleavage product may modulate this effect. Further studies will be required to determine if caspase cleavage of LANA plays other roles in promoting KSHV infection or KSHV disease pathogenesis.

## Materials and Methods

### Cell culture and reagents

BC-3 (American Type Culture Collection (ATCC), Manassas, VA)[52], CA46 (ATCC), THP-1 (ATCC), and BCBL-1 cells (NIAID AIDS Research and Reagent Program, Rockville, MD) were cultured in RPMI 1640 medium (Life Technologies, Carlsbad, CA) with 15% heat-inactivated fetal calf serum (HyClone,Waltham, MA), 1% penicillin streptomycin glutamine (1000 units/ml penicillin, 10000 μg ml^-1^ streptomycin, 29.2 mg/ml L-glutamine) (Life Technologies), and supplemented with 20μM β-mercaptoethanol (Sigma). HEK293T (ATCC) and Hep-3B cells (ATCC) were cultured at 37°C in DMEM (Life Technologies) with 10% heat-inactivated fetal calf serum and 1% penicillin streptomycin glutamine (Life Technologies). Hydrogen peroxide 30% (H_2_O_2_) (Fisher Scientific, Pittsburg, PA) was diluted fresh into sterile PBS prior to use. Stocks of Bay-11 (Sigma), (Sigma), pan-caspase inhibitor (ZVAD-FMK (designated ZVAD in the text)) and negative control inhibitor (Z-FA-FMK) (Sigma and Calbiochem, Darmstadt, Germany), caspase-3/7 inhibitor (5-[(S)-(+)-2-(Methoxymethyl)pyrrolidino]sulfonylisatin) (Calbiochem) and caspse-1 inhibitor IV (Ac-YVAD-AOM) (Calbiochem) were dissolved in 100% DMSO (Sigma). When screening for caspase cleavage of LANA, a caspase family of enzymes from Calbiochem were used (includes caspase-1, 2,3,6,7,8,9 and 10).

### Induction of oxidative stress and apoptosis

BCBL-1 and BC-3 cells were pelleted and resuspended in media at 400,000 live cells ml^-1^ and treated with PBS and/or DMSO (0.25% as a vehicle controls), H_2_O_2_ (final 10–100 μM), Bay-11 or etoposide. Cell viability was assessed by trypan blue (Life Technologies, Grand Island, NY) exclusion or by measuring total ATP with the CellTiterGlo assay (Promega, Madison, WI).

### Western blot

Nuclear and cytoplasmic extracts were harvested using NE-PER Nuclear and Cytoplasmic Extraction Reagents kit (Pierce, Rockford, IL) in the presence of protease inhibitors (Halt Protease Inhibitors Cocktail Kit, Pierce) and 5 mM EDTA. Protein concentrations were determined using the BCA assay (Pierce) and samples were separated on a 4–12% NuPAGE gel, and transferred to a nitrocellulose membrane by iBlot (Life technologies). Membranes were blocked in 5% non-fat dry milk in tris-buffered saline and 0.5% Tween 20 and probed with mouse-anti LANA (Leica Biosystems, Buffalo Grove, IL), mouse anti-β-actin (Sigma), or rabbit anti-FLAG (Sigma) primary antibodies. Blots were then incubated with appropriate secondary antibodies conjugated to alkaline phosphatase and visualized using stabilized Western Blue substrate (Promega) or with a goat anti-mouse or rabbit IR700 or IR800 secondary antibody (diluted 1:10,000) as indicated. Membranes exposed to LI-COR secondary antibodies were scanned using a LI-COR Odyssey infrared scanner. Images were analyzed using Image Studio 2.1 software.

### Production of FLAG-tagged LANA and *in vitro* caspase cleavage of FLAG-LANA

For studies on caspase cleavage of LANA, a FLAG-tagged LANA expression vector was prepared. Viral DNA from KSHV-infected BCBL-1 cells was used as a template for PCR amplification. Full-length ORF73 (LANA) gene was amplified by PCR with specific primers: ORF73F- CAT GAA TTC ATG GCG CCC CCG GGA ATG CGC, and ORF73R- CAT AAG CTT TGT CAT TTC CTG TGG AGA GTC containing EcoRI and HindIII restriction sites at the 5' and 3' ends, respectively. The amplified products were digested with EcoRI and HindIII (New England BioLabs, Ipswich, MA) and inserted into the EcoRI and HindIII restriction sites of a FLAG-tagged expression vector, pCMV-Tag2B (Agilent Technologies, Santa Clara, CA). Three additional FLAG-tagged LANA expression vectors were prepared containing mutations in putative caspase cleavage sites. Aspartate-to-alanine mutations were made at amino acid position 53 (GAC->GCC) and 878 (GAT->GCT) of the BCBL-1 LANA amino acid sequence (GENBANK accession number U93872). One each was constructed to contain a mutation at either of two putative caspase cleavage sites and one contained a mutation at both cleavage sites. Plasmids expressing mutated caspase cleavage sites of LANA were constructed using the PCR-based QuikChange site-directed mutagenesis kit (Stratagene, La Jolla, CA) according to the manufacturer’s protocol. DNA was amplified by PCR with complimentary primer pairs and wild type expression plasmid pCMV-LANA as the template. The complimentary primer sequences for the LANA-N terminal mutation (Asp to Ala mutation) were as follows: WT: GTCGCCGACTCCGTCGACGGCCGGGAATGTGG LANANMF2 (5’-GTCGCCGACTCCGTCGCCGGCCGGGAATGTGG -3’) and LANANMR2 (5’-CCACATTCCCGGCCGGCGACGGAGTCGGCGAC -3’). The complimentary primer sequences for the LANA-C terminal mutation (Asp-to-Ala mutation) are as follows: LANACMF 5’- GGACGAAATGGAAGTGGCTTACCCTGTTGTTAGCAC-3’ and LANACMR 5’-GTGCTAACAACAGGGTAAGCCACTTCCATTTCGTCC-3’. The PCR reactions were performed for 18 cycles at 95°C for 30 seconds, 55°C for 1 min and 68°C for 8 min. The PCR products were incubated with DpnI enzyme (New England Biolabs) at 37°C for 2 hours to digest the parental vector. The mutated plasmid DNA was used to transform XL-1 Blue competent cells (Stratagene. La Jolla, CA). All plasmid constructs were confirmed by DNA sequencing and then purified with Qiagen Maxiprep kit (Qiagen, Valencia, CA).

HEK293T cells were plated at 160,000 live cells ml^-1^ and cultured overnight at 37°C. Cells were transfected using Fugene 6 transfection reagent (Promega) with FLAG-tagged LANA-pCMV wild-type, mutant-LANA-pCMV, or empty FLAG-pCMV vectors. Forty hours post-transfection, nuclear extracts containing FLAG-LANA were harvested. Nuclear extracts containing FLAG-LANA were treated with caspases (Calbiochem) and analyzed by western blot to assess cleavage of LANA.

### Synthesis and caspase cleavage of LANA peptides

The webserver CasCleave was used to identify potential caspase cleavage sites in the LANA amino acid sequence. Three peptides containing one likely caspase cleavage site from the LANA sequence, were synthesized (New England Peptide, Gardner, MA). The three peptides had the following sequences: RKRNRSPERCDLGDDLHLQ; LP-Nterm, RRKHVADSVD*GRECGPH; and LP-Cterm, SSSEDEMEVD*YPVVSTHE, the asterisk indicates the predicted site of cleavage). To identify if these were functional caspase cleavage sites, 1 mM peptide was treated with caspases as described in cleavage buffer (100 mM NaCl, 50 mM HEPES, 1 mM EDTA, 10 mM DTT, 0.1% CHAPS, 10% glycerol) in the presence or absence of 50 μM ZVAD overnight at 37°C. The cleavage reaction was stopped with 5 M guanidine + 0.1% trifluoroacetic acid (TFA) (Pierce). The reaction mixture was then analyzed by reverse phase high performance liquid chromatography (RP-HPLC) on a 1200 Series HPLC (Agilent Technologies, Santa Clara, CA) connected to an electrospray ionization mass spectrometer (Agilent Technologies) used to determine the molecular weight of the intact and cleaved peptide products. Samples were separated on a Vydac C18 column (Agilent Technologies) using a water/acetonitrile gradient at a flow rate of 0.5 ml min/min. Solvent A was deionized water with 0.05% TFA and solvent B was acetonitrile with 0.025% TFA and 0.05% formic acid. The column was equilibrated at 0.5 ml/min with 95% solvent A and 5% solvent B for 10 minutes prior to each injection. After injection, solvent A was increased to 25% over the next 25 minutes and then increased to 95% solvent B for 2 minutes and brought back to 95% solvent A in the next two minutes. Peptide elution was monitored with a diode array detector set at 205 nm and 276 nm and by ionization mass spectrometry (drying gas 12 l min^-1^, nebulizer 35 psig, drying gas 350, voltage 3000) set to analyze a mass range of 450–1200 Daltons.

### Caspase activity assays

Caspase-1 and caspase-3 activity was measured based on the cleavage of a caspase-1 substrate (Ac-YEVD-pNa, Sigma) or caspase-3 substrate (Ac-DEVD-pNa, Sigma), respectively (Caspase 3 Activity Assay Kit, Colorimetric; Sigma). In a clear flat-bottom 96-well plate, cleavage buffer (50 mM HEPES, pH 7.4, 100 mM NaCl, 0.1% CHAPS, 10 mM DTT, 1 mM EDTA, 10% glycerol), LANA peptides (tested as competitive inhibitors of caspases) and the colorimetric caspase substrate were added to the plate in that order. The reaction was started with the addition of caspase-3 (Sigma; 0.25 μg ml^-1^) or caspase-1 (Calbiochem; 0.5 units μl^-1^) and activity was monitored and the rate of reaction over the 30-minute incubation period determined.

### Analysis of poly ADP Ribose polymerase (PARP) cleavage in HEP3B cells and interleukin-1beta (IL-1β) production from THP-1 cells

HEP3B cells were used to determine if peptides corresponding to the LANA caspase cleavage sites affected PARP cleavage by caspases upon induction of apoptosis with etoposide. For these experiments LP-Nterm and LP-Cterm were prepared with an N-terminal nine amino acid Tat delivery sequence RKKRRQRRR [29] in order to increase cellular uptake of the peptides. HEP3B cells were plated in 6 well plates (3 ml) at 5 x 10^5^ cells ml^-1^. Two days later the cells were pretreated for two hrs with peptides or the positive control caspase inhibitor ZVAD (Sigma). Cells were then treated with either DMSO vehicle or 50μM etoposide to induce PARP cleavage and apoptosis. Cell lysates were prepared 16 h later in M-per mammalian lysate reagent (Thermo) and protein determined using the BCA assay reagent (Pierce). Samples were analyzed by separating equal amounts of protein on a 4–12% Nupage gel (Invitrogen). Protein was transferred by iblot (Invitrogen) and then probed with a rabbit antibody to cleaved PARP (Cell Signaling) and a mouse antibody to β-actin (Sigma). Proteins were visualized following exposure of blots to either alkaline phosphate-linked rabbit and mouse secondary antibodies or fluorescent-labeled anti-mouse and anti-rabbit antibodies (LI COR Biosciences, Lincoln, NE). Quantification of the PARP and β-actin bands was performed using ImageStudio2 (LI COR Biosciences).

THP-1 cells, a monocyte derived cell line, were used to assess the effects of cell permeable peptide derivatives of LP-Nterm and LP-Cterm containing the N or C-terminal cleavage sites, respectively, on IL-1β production without and with LPS treatment. We also examined the effects of transfected WT and mutant forms of LANA on IL-1β production. Cells were plated at 500,000 cells ml^-1^ in 12 well plates (1 ml) and matured by treating with 200 nM TPA. The next day the media was removed and fresh media was added. The cells were then pretreated with control (DMSO), ZVAD (to inhibit caspase activity), or the cell permeable peptides (Tat-LP-Nterm and Tat-LP-Cterm) containing either the N or C terminal cleavage site identified in LANA. Two hours later the cells were either treated with PBS (control) or lipopolysaccharide (LPS) (Sigma, St. Louis MO.) at 100 ng/ml to induce the inflammasome response. After 20 h the media was sampled and assayed for IL-1β by ELISA following the manufacturers instructions (R&D Systems, Minneapolis, MN). The cells adhered to the plate were then washed three times with PBS and lysed with a constant volume of lysis buffer to assess total protein as a measure of any effect on cell viability. Protein was separated by LDS-PAGE, transferred to nitrocellulose and probed with a mouse primary antibody to IL-1β (Cell Signaling) or a rabbit primary antibody to caspase-1 (Cell Signaling). Blots were then incubated with appropriate secondary antibodies (goat anti-mouse or rabbit IR700 or IR800 secondary antibody, diluted 1:10,000) as indicated and analyzed using the LiCor system. For IL-1β production from peptide treated cells the statistical comparisons were done using a two-tailed Student’s paired t test. For LANA transfection of THP-1 cells, the cells were plated at 400,000 cells ml^-1^ in 12 well plates and matured with TPA (200 nM) overnight. The media was replaced and the cells were transfected with the WT LANA and mutant LANA plasmids (250 ng DNA per well) using fugene-6 as described above. After 24 h transfection cells were treated with PBS (control) or LPS (100 ng/ml) to induce the inflammasome response. Media was harvested after 24 and 48 h to assess IL-1β levels and cell lysates were prepared to look at various inflammasome related proteins. For IL-1β production from transfected cells the statistical comparisons were performed using the raw data and using the two-tailed Student’s paired t test.

## Supporting Information

S1 Fig FLAG-LANA treated for 16 hrs at 37°C with 0.15 units caspases μg^-1^ protein and probed with the Leica antibody to LANA, which is directed toward the C-terminal 265 amino acids. The large fragment predicted to be generated by cleavage at the N-terminus (LANAcp2) and the large fragment predicted to be truncated at the N and C-terminus (LANAcp5) is indicated.
(TIF)

S2 Fig Inducers of oxidative stress and apoptosis lead to an increase of full-length LANA and also the appearance of faster migrating forms of LANA. BCBL-1 cells were treated with increasing concentrations, Bay-11 for 24 hours (A-B) or etoposide for 24 hours (C-D),. Nuclear and cytoplasmic extracts were prepared and LANA protein expression was analyzed by western blot (alkaline phosphatase system) using a mouse monoclonal antibody to LANA. Native full length LANA (based on molecular weight) (LANA-fl) as well as lower molecular weight forms of LANA designated as LANAcp2, LANAcp3 and LANAcp5 are indicated. Tυβυλιν is shown below each blot for LANA as a loading control.
(TIF)

S3 Fig Mutation of both LANA caspase cleavage sites leads to increased IL-1β production. THP-1 cells were matured over night by treatment with TPA and then transiently transfected with plasmid Vector control or FLAG-tagged forms of WT-LANA, LANA-NMUT, LANA-CMUT or LANA-DM. The next day cells were treated with vehicle control (cell culture media) and the level of IL-1β in the supernatants measured 20 hrs later. Cell extracts were also made for protein analysis and immunoblots. (A) IL-1β levels as determined by ELISA following transfection (not treated with LPS). (B) Immunoblot for cleaved caspase-1 and actin showing the relative levels of active casapse-1 in transfected cells compared to vector control as determined by the LiCor system. Data shown in (A) are the average +/- the standard deviation from 4 independent experiments. * *P*< 0.05, *** *P*< 0.005 for two-tailed Student’s t-test.
(TIF)

S4 Fig Caspase cleavage sites of LANA do not affect IL-18 production. Cell permeable peptides with an N-terminal cellular HIV-1 Tat delivery sequence RKKRRQRRR containing the N-terminal caspase cleavage site of LANA (T-LP-Nterm) or the C-terminal caspase cleavage site of LANA (T-LP-Cterm) were tested as potential inhibitors of IL-18 production following treatment of THP-1 cells with LPS. ZVAD was used as a positive caspase inhibitor control. (A) Effect of ZVAD (50 μM), T-LP-Nterm (50 μM) and T-LP-Cterm (50 μM) on IL-18 in the supernatant from THP-1-treated cells. Δata is from three separate experiments. (B) Mutation of LANA caspase cleavage sites does not affect IL-18 production. THP-1 cells were matured overnight by treatment with TPA and then transiently transfected with FLAG-tagged forms of WT-LANA, LANA-NMUT, LANA-CMUT or LANA-DM. The next day cells were treated with vehicle control (cell culture media) and the level of IL-18 in the supernatants measured 20 hrs later. Data is from the average of two experiments with similar results.
(TIF)
