# Supplementary figures and images for: Identification of Caspase Cleavage Sites in KSHV Latency-Associated Nuclear Antigen and Their Effects on Caspase-Related Host Defense Responses
Source: PLoS Pathog. 2015 Jul 28;11(7):e1005064. doi: 10.1371/journal.ppat.1005064 (PMC4517896; doi:10.1371/journal.ppat.1005064)

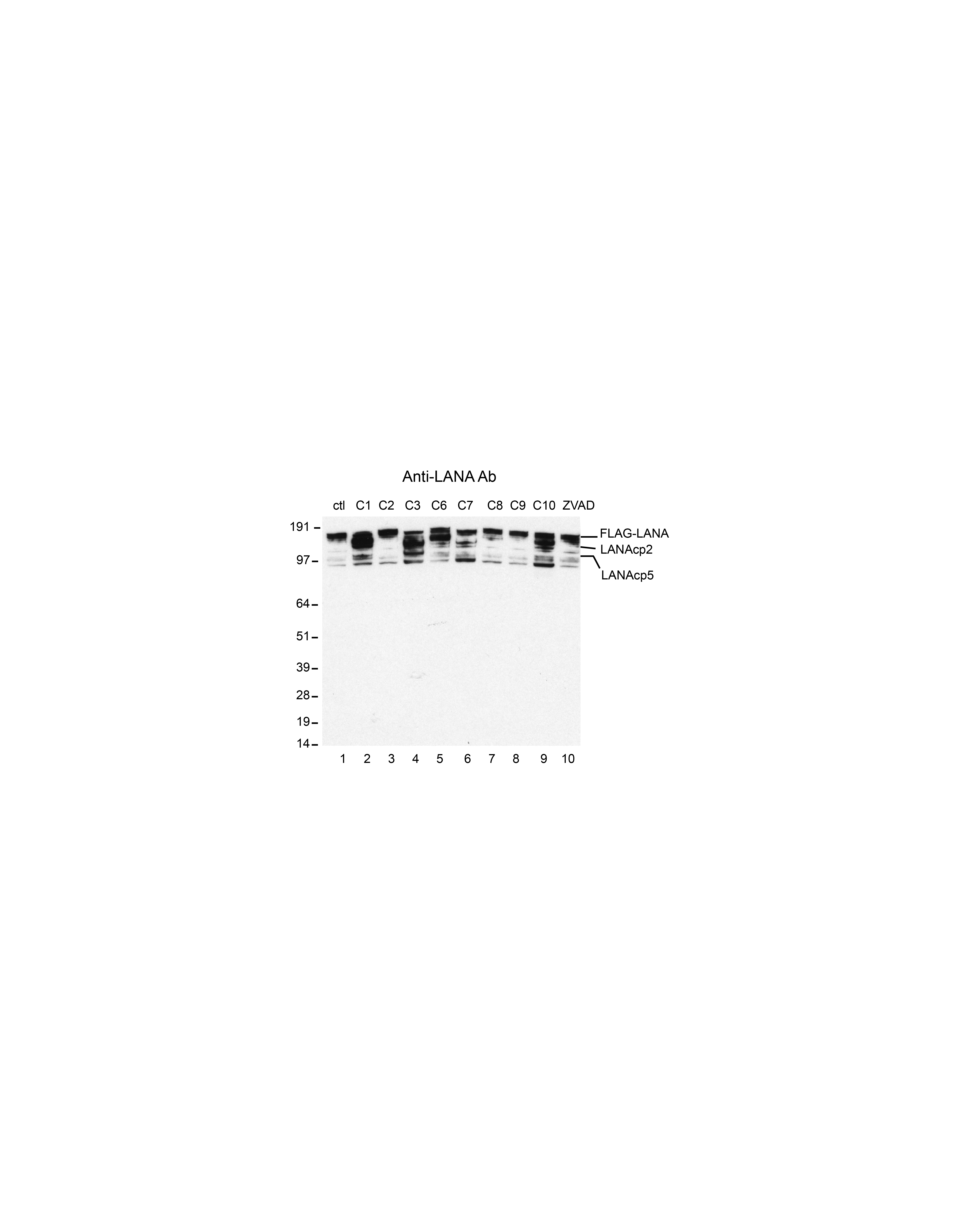

Supplement: S1 Fig — The large fragment predicted to be generated by cleavage at the N-terminus (LANAcp2) and the large fragment predicted to be truncated at the N and C-terminus (LANAcp5) is indicated. (TIF) [file ppat.1005064.s001.tif]

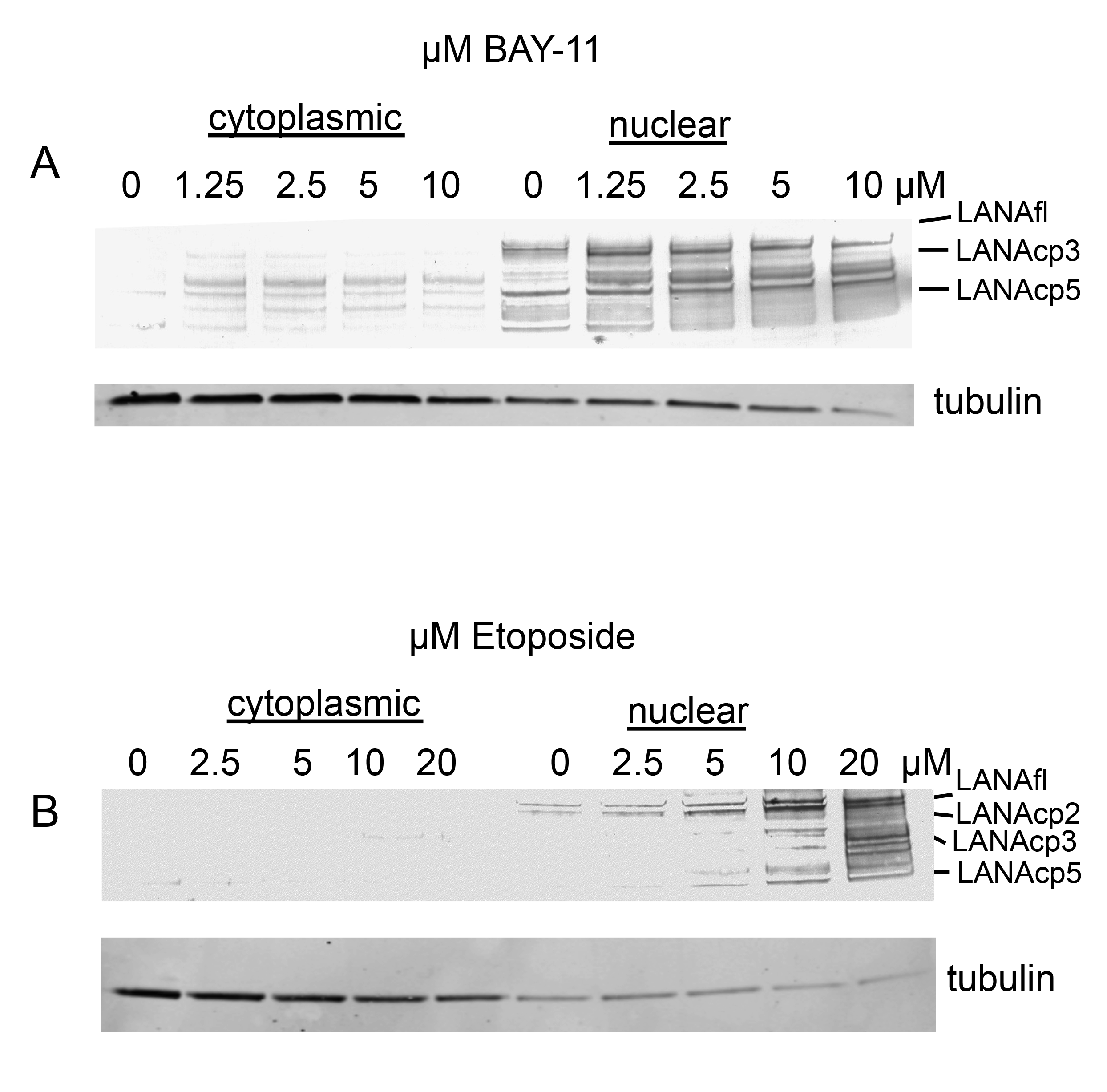

Supplement: S2 Fig — BCBL-1 cells were treated with increasing concentrations, Bay-11 for 24 hours (A-B) or etoposide for 24 hours (C-D),. Nuclear and cytoplasmic extracts were prepared and LANA protein expression was analyzed by western blot (alkaline phosphatase system) using a mouse monoclonal antibody to LANA. Native full length LANA (based on molecular weight) (LANA-fl) as well as lower molecular weight forms of LANA designated as LANAcp2, LANAcp3 and LANAcp5 are indicated. Tυβυλιν is shown below each blot for LANA as a loading control. (TIF) [file ppat.1005064.s002.tif]

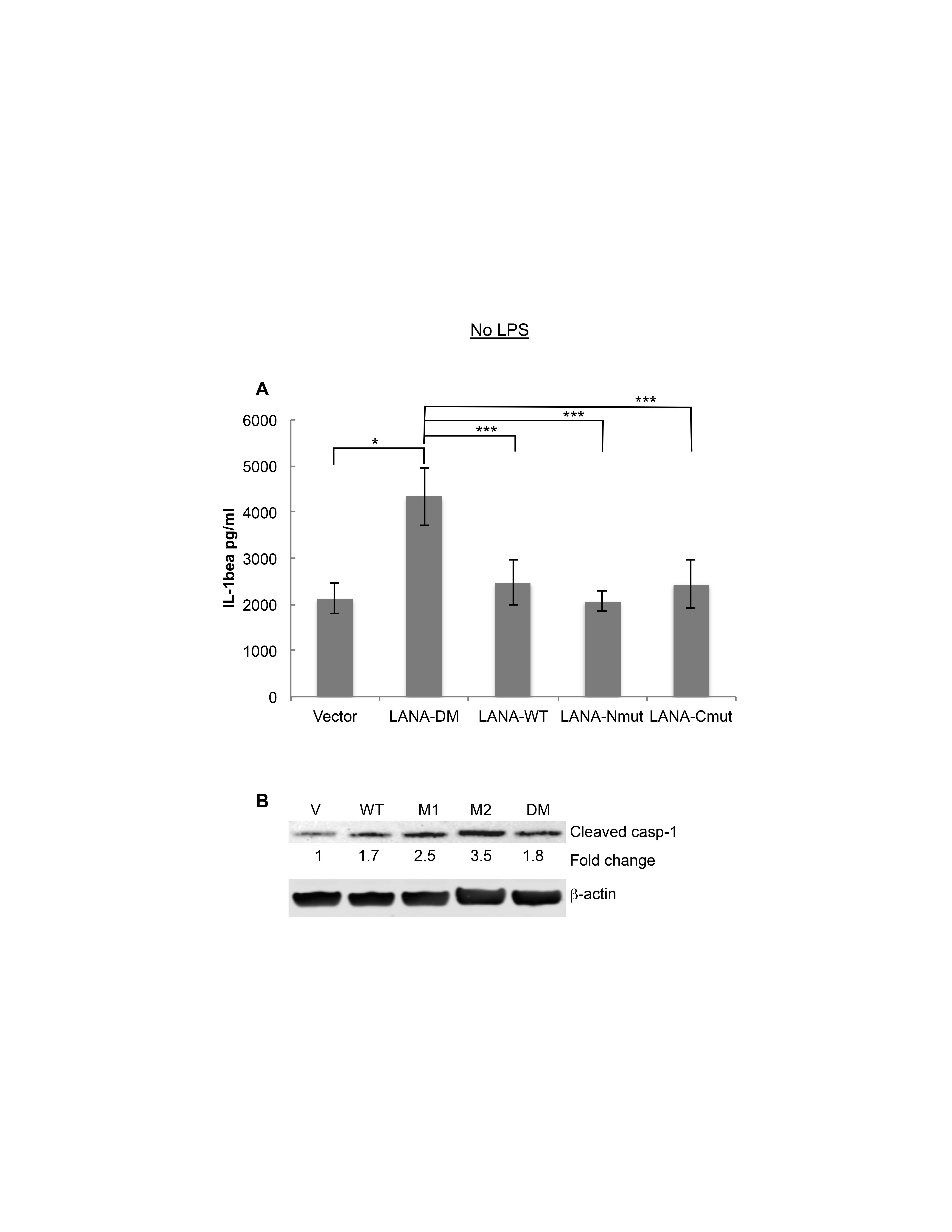

Supplement: S3 Fig — THP-1 cells were matured over night by treatment with TPA and then transiently transfected with plasmid Vector control or FLAG-tagged forms of WT-LANA, LANA-NMUT, LANA-CMUT or LANA-DM. The next day cells were treated with vehicle control (cell culture media) and the level of IL-1β in the supernatants measured 20 hrs later. Cell extracts were also made for protein analysis and immunoblots. (A) IL-1β levels as determined by ELISA following transfection (not treated with LPS). (B) Immunoblot for cleaved caspase-1 and actin showing the relative levels of active casapse-1 in transfected cells compared to vector control as determined by the LiCor system. Data shown in (A) are the average +/- the standard deviation from 4 independent experiments. * P< 0.05, *** P< 0.005 for two-tailed Student’s t-test. (TIF) [file ppat.1005064.s003.tif]

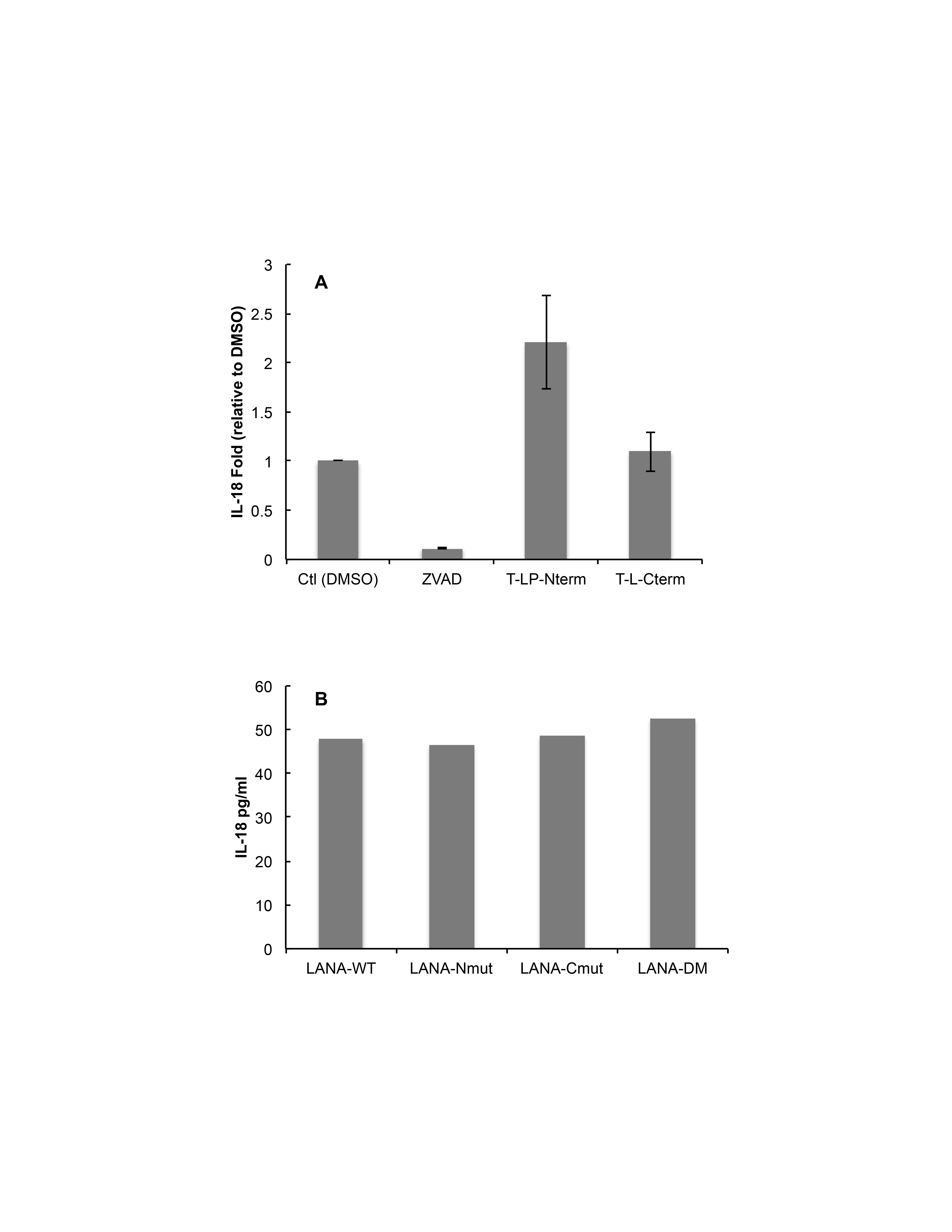

Supplement: S4 Fig — Cell permeable peptides with an N-terminal cellular HIV-1 Tat delivery sequence RKKRRQRRR containing the N-terminal caspase cleavage site of LANA (T-LP-Nterm) or the C-terminal caspase cleavage site of LANA (T-LP-Cterm) were tested as potential inhibitors of IL-18 production following treatment of THP-1 cells with LPS. ZVAD was used as a positive caspase inhibitor control. (A) Effect of ZVAD (50 μM), T-LP-Nterm (50 μM) and T-LP-Cterm (50 μM) on IL-18 in the supernatant from THP-1-treated cells. Δata is from three separate experiments. (B) Mutation of LANA caspase cleavage sites does not affect IL-18 production. THP-1 cells were matured overnight by treatment with TPA and then transiently transfected with FLAG-tagged forms of WT-LANA, LANA-NMUT, LANA-CMUT or LANA-DM. The next day cells were treated with vehicle control (cell culture media) and the level of IL-18 in the supernatants measured 20 hrs later. Data is from the average of two experiments with similar results. (TIF) [file ppat.1005064.s004.tif]
